# The woody plant-degrading pathogen *Lasiodiplodia theobromae* effector LtCre1 targets the grapevine sugar-signaling protein VvRHIP1 to suppress host immunity

**DOI:** 10.1093/jxb/erad055

**Published:** 2023-02-15

**Authors:** Qikai Xing, Xiangui Zhou, Yang Cao, Junbo Peng, Wei Zhang, Xuncheng Wang, Jiahong Wu, Xinghong Li, Jiye Yan

**Affiliations:** Beijing Key Laboratory of Environment Friendly Management on Fruits Pests in North China, Institute of Plant Protection, Beijing Academy of Agriculture and Forestry Sciences, Beijing 100097, China; State Key Laboratory of Protein and Plant Gene Research, School of Advanced Agricultural Sciences and School of Life Sciences, Peking-Tsinghua Center for Life Sciences, Peking University, 100871 Beijing, China; Beijing Key Laboratory of Environment Friendly Management on Fruits Pests in North China, Institute of Plant Protection, Beijing Academy of Agriculture and Forestry Sciences, Beijing 100097, China; Beijing Key Laboratory of Environment Friendly Management on Fruits Pests in North China, Institute of Plant Protection, Beijing Academy of Agriculture and Forestry Sciences, Beijing 100097, China; Beijing Key Laboratory of Environment Friendly Management on Fruits Pests in North China, Institute of Plant Protection, Beijing Academy of Agriculture and Forestry Sciences, Beijing 100097, China; Beijing Key Laboratory of Environment Friendly Management on Fruits Pests in North China, Institute of Plant Protection, Beijing Academy of Agriculture and Forestry Sciences, Beijing 100097, China; Beijing Key Laboratory of Environment Friendly Management on Fruits Pests in North China, Institute of Plant Protection, Beijing Academy of Agriculture and Forestry Sciences, Beijing 100097, China; Beijing Key Laboratory of Environment Friendly Management on Fruits Pests in North China, Institute of Plant Protection, Beijing Academy of Agriculture and Forestry Sciences, Beijing 100097, China; Beijing Key Laboratory of Environment Friendly Management on Fruits Pests in North China, Institute of Plant Protection, Beijing Academy of Agriculture and Forestry Sciences, Beijing 100097, China; Sichuan Agricultural University, China

**Keywords:** Fungal effector, grapevine, *Lasiodiplodia theobromae*, plant immunity, plant–pathogen interaction, sugar signaling, susceptibility, *Vitis vinifera*

## Abstract

*Lasiodiplodia theobromae* is a causal agent of Botryosphaeria dieback, which seriously threatens grapevine production worldwide. Plant pathogens secrete diverse effectors to suppress host immune responses and promote the progression of infection, but the mechanisms underlying the manipulation of host immunity by *L. theobromae* effectors are poorly understood. In this study, we characterized *LtCre1*, which encodes a *L. theobromae* effector that suppresses BAX-triggered cell death in *Nicotiana benthamiana*. RNAi-silencing and overexpression of *LtCre1* in *L. theobromae* showed impaired and increased virulence, respectively, and ectopic expression in *N. benthamiana* increased susceptibility. These results suggest that LtCre1 is as an essential virulence factor for *L. theobromae*. Protein–protein interaction studies revealed that LtCre1 interacts with grapevine RGS1-HXK1-interacting protein 1 (VvRHIP1). Ectopic overexpression of *VvRHIP1* in *N. benthamiana* reduced infection, suggesting that VvRHIP1 enhances plant immunity against *L. theobromae*. LtCre1 was found to disrupt the formation of the VvRHIP1–VvRGS1 complex and to participate in regulating the plant sugar-signaling pathway. Thus, our results suggest that *L. theobromae* LtCre1 targets the grapevine VvRHIP1 protein to manipulate the sugar-signaling pathway by disrupting the association of the VvRHIP1–VvRGS1 complex.

## Teaser

The *Lasiodiplodia theobromae*LtCre1 effector disrupts the formation of the VvRHIP1–VvRGS1 complex in grapevine, which interferes with sugar signaling and suppresses the expression of genes related to plant immunity

## Introduction

Grapevine trunk diseases (GTDs) pose a severe threat to grape production and the wine industry worldwide (Bertsch *et al.*, 2013; Gramaje *et al.*, 2018; Mondello *et al.*, 2018). The fungi causing GTDs, which generally infect grapevines through pruning wounds and natural openings, colonize the vascular tissue system, retarding growth and leading to wood necrosis or cankers, ultimately resulting in plant death (Makatini *et al.*, 2014; Moisy *et al.*, 2017; Spagnolo *et al.*, 2017; Songy, *et al.*, 2019). However, the exact roles of the GTD fungi in these disease symptoms remain unclear. *Lasiodiplodia theobromae* (family *Botryosphaeriaceae*), which has been reported to attack ~500 fruit crops and woody trees (Phillips *et al.*, 2013; Cipriano *et al.*, 2015; Gramaje *et al.*, 2016), is one of the most aggressive and widely distributed vascular pathogens of grapevines in particular (Úrbez-Torres, 2011; Yan *et al.*, 2013; Yang *et al.*, 2017; Gramaje *et al.*, 2018). To develop efficient strategies for GTD management, it is necessary to better understand the molecular bases of the grapevine–*L. theobromae* interaction. The publication of whole-genome sequences of *L. theobromae* offers an excellent resource for studying the molecular biology of GTD fungi in woody plants (Chethana *et al.*, 2016;  Yan *et al.*, 2018).

The plant innate immune system has evolved two layers of pathogen defense: pathogen-associated molecular pattern (PAMP)-triggered immunity (PTI) and effector-triggered immunity (ETI) (Jones and Dangl, 2006; Boller and Felix, 2009; Boller and He, 2009; Zipfel, 2008; Dodds and Rathjen, 2010; Dangl *et al.*, 2013; PAMP is also referred to as MAMP, for microbe-associated molecular pattern). PTI is mediated by pattern-recognition receptors at the plasma membrane and normally triggers basal defense responses, including the production of reactive oxygen species (ROS), callose deposition, the accumulation of antimicrobial components, and the regulation of pathogenesis-related genes (Jones and Dangl, 2006; Zipfel, 2008; Boller and Felix, 2009; Dodds and Rathjen, 2010). Meanwhile, pathogen effectors have been reported to target diverse plant pathways at various levels to either suppress host immunity or manipulate host metabolism, thus promoting pathogen pathogenicity (de Jonge *et al.*, 2011; Lo Presti *et al.*, 2015; Tomczynska *et al.*, 2018; Yang *et al.*, 2019; Du *et al.*, 2021; Ma *et al.*, 2021). To combat this, ETI is triggered by the recognition by plant intracellular disease resistance (R) proteins of specific pathogen effectors and induces a rapid and robust hypersensitive response (HR) at the infected site to restrict pathogen invasion (Jones and Dangl, 2006; Lo Presti *et al.*, 2015; Jones *et al.*, 2016; Zhou and Zhang, 2020). Studies of the molecular mechanisms of pathogen effectors have not only elucidated pathogen infection processes but have also identified novel components of plant immunity.

In particular, sugars such as glucose, fructose, and sucrose have been recognized as multifaceted metabolites and signaling molecules that not only affect plant growth and development but also plant immune responses (Bolouri-Moghaddam *et al.*, 2010; Sheen, 2014; Sakr *et al.*, 2018; Hennion *et al.*, 2019; Salmon *et al.*, 2020). In Arabidopsis, REGULATOR OF G-PROTEIN SIGNALING PROTEIN 1 (AtRGS1), HEXOKINASE 1 (AtHXK1), and SNF1-RELATED KINASE 1/TARGET OF RAPAMYCIN (SnRK1/TOR) act as glucose sensors and are involved in the regulation of sugar signaling (Grigston *et al.*, 2008). Treatment of Arabidopsis with D-glucose and the potent elicitor of plant immune responses flg22 causes endocytosis of AtRGS1, leading to the activation of heterotrimeric G protein-mediated sugar signaling (Urano *et al.*, 2012; Fu *et al.*, 2014; Tunc-Ozdemir *et al.*, 2016). Arabidopsis RGS1-HXK1-INTERACTING PROTEIN 1 (AtRHIP1), which provides the physical scaffold for AtRGS1 and AtHXK1, also plays a pivotal role in glucose-regulated gene expression (Huang *et al.*, 2015). In addition, glucose sensors are known to regulate plant defense responses through sugar-signaling pathways during plant–pathogen interactions (Moore *et al.*, 2003; Sheen, 2014). A previous study has shown that AtRGS1, which stabilizes G proteins in the FLS2 receptor complex, plays a significant role in flg22- and chitin-induced ROS production and in defense-gene expression, and enhances resistance to *Pseudomonas syringae* (Liang *et al.*, 2018). Overexpression of the apple hexokinase gene *MdHXK1* activates salicylic acid (SA) signaling and enhances disease resistance to *Botryosphaeria dothidea* infection by increasing the contents of superoxide and hydrogen peroxide in apple calli, fruit, and leaves (Yu *et al.*, 2022). However, the specific mechanisms underlying the manipulation of plant sugar signaling by plant pathogens remain largely unknown.

Studies of the genomes of GTD fungi have shown that a wide array of potential virulence factors play roles in pathogenicity during pathogen infection, such as secondary metabolites, secretory proteins, and cell wall-modifying enzymes (Antonielli *et al.*, 2014; Blanco-Ulate *et al.*, 2013; Malapi-Wight *et al.*, 2015; Morales-Cruz *et al.*, 2015; Paolinelli-Alfonso *et al.*, 2016; Yan *et al.*, 2018). In the *L. theobromae* genome, small, secreted proteins carrying signal peptides, accounting for ~4.3% of the total number of predicted proteins in the whole genome, have been identified as candidates (Yan *et al.*, 2018; Xing *et al.*, 2020). At least 300 of these small proteins are also cysteine-rich and are thus predicted to be effector candidates (Yan *et al.*, 2018). Transcriptome profiling during *L. theobromae*–grapevine interactions and HR-inhibition assays in *Nicotiana benthamiana* have suggested that a subset of putative effectors play roles during early colonization and pathogenesis (Yan *et al.*, 2018; Xing *et al.*, 2020). In addition, the first cloned *L. theobromae* effector gene, *LtEPG1*, which encodes an endopolygalacturonase protein, promotes virulence and acts as a MAMP during the infection process (Chethana *et al.*, 2020). However, little else is known about the molecular mechanisms underlying the interactions between candidate effectors and host targets in *L. theobromae*.

We have previously reported that a set of effector genes are induced at the early infection stage of *L. theobromae* (Yan *et al.*, 2018). In this current study, one of the putative secreted cysteine-rich effectors (here named LtCre1), which possesses a RXLR domain, was chosen as a representative *L. theobromae* effector to assess its effects on grapevine immunity. We show that LtCre1 suppresses the BAX-triggered HR, and that expression of *LtCre1* is strongly up-regulated at the early stages of infection. The expression of *LtCre1* is crucial for the full virulence of *L. theobromae* and it promotes leaf colonization when ectopically expressed in *N. benthamiana*. A *Vitis vinifera* RGS1-HXK1-interacting protein 1 (VvRHIP1) that is associated with the sugar-signaling pathway is identified as a target of LtCre1. We demonstrate that VvRHIP1 acts as a positive regulator of grapevine resistance to *L. theobromae*. Based on our results, we propose that LtCre1 probably manipulates plant immunity and the sugar-signaling pathway by disrupting the VvRHIP1–VvRGS1 complex.

## Materials and methods

### Plant growth conditions and pathogen inoculation assays

The grapevine cultivar *Vitis vinifera* var. ‘Summer Black’, which is susceptible to *Lasiodiplodia theobromae*, was cultivated in a greenhouse at the Beijing Academy of Agriculture and Forestry Sciences, Beijing, China. Stem inoculations were performed as previously described by Yan *et al.* (2018). The *L. theobromae* isolate CSS-01s (wild-type, WT) was cultured on potato dextrose agar medium (potato 200 g l^−1^, dextrose 20 g l^−1^, and agar 20 g l^−1^) at 26 °C for 14−21 d, and conidia spores were collected for inoculation. Stems of *V. vinifera* were wounded using a 4-mm cork-borer (2 mm deep), and the wounds were inoculated with conidial suspension at a concentration of 1 × 10^6^ conidia ml^−1^ in 0.02% Silwet L-77. The infected stems were kept in an inoculation room under a 16/8-h light/dark photoperiod (130 µmol m^−2^ s^−1^) at 26 °C with a relative humidity (RH) of 90%. Lesion lengths were measured 5−7 d post-inoculation. To investigate the expression patterns of *LtCre1* at the early infection stages of *L. theobromae*, stem phloem samples were taken within 3.5–4.0 cm of the wound point at 12, 24, 36, and 48 h post-inoculation. The samples were immediately frozen in liquid nitrogen and stored at –80 °C.

Seedlings of *Nicotiana benthamiana* were grown in a soil mixture under a 16/8-h light/dark cycle at 26 °C in a greenhouse, and 4-week-old plants were used for co-immunoprecipitation (CoIP) and bimolecular fluorescence complementation (BiFC) assays. For pathogen infection assays, the abaxial surfaces of detached leaves from 4-week-old plants were inoculated with 10 µl of conidial suspension at a concentration of 1 × 10^6^ conidia ml^−1^. The inoculated leaves were placed at 26 °C with high humidity for 4−5 d, and disease severity was evaluated by measuring lesion diameters. At 12 h post-inoculation, leaves were harvested for subsequent isolation of RNA.

### Transformation of *N. benthamiana*

The coding sequence (CDS) of *LtCre1* without the predicted secretory signal peptide (SP) was amplified from the cDNA of *L. theobromae* using the primers CreG-BIF and CreG-SIR (Supplementary Table S1). The amplified CDS was cloned into the vector Cam35S:GFP (Liu *et al.*, 2016) using *Bam*H I and *Sal* I to construct the overexpression vector. The resulting recombinant vector Cam35S:LtCre1^ΔSP^-GFP was introduced into *Agrobacterium tumefaciens* strain EHA105 for *N. benthamiana* leaf disc transformation as previously described (Gallois and Marinho, 1995).

### Genetic transformation of *L. theobromae*

To generate the overexpression vector, the full-length CDS of *LtCre1* was subcloned into the *Hin*d III-*Pst*I sites in a modified pBluescript II KS vector (Yan *et al.*, 2018). For RNA interference (RNAi), a 213-bp fragment of *LtCre1* was amplified and ligated into the pMD18-T vector (TaKaRa) with the opposite orientation. The hairpin-structured fragment was then sequentially ligated into the *Hin*d III-*Eco*R I sites in the modified pBluescript II KS vector. *Lasiodiplodia theobromae* was stably transformed using polyethylene glycol (PEG)-mediated protoplast transformation (Yan *et al.*, 2018). Positive transformants were screened using PCR and confirmed with quantitative real-time (qRT-)PCRusing the gene-specific primers Cre-qF and Cre-qR (Supplementary Table S1).

### Gene expression analysis

Total RNA was isolated from the stem phloem samples using an EASYspin Plus Complex Plant RNA Kit (Aidlab, Beijing, China), from the inoculated *N. benthamiana* leaves using a *TransZol* Plant RNA Kit (TransGen Biotech, Beijing, China), and from *L. theobromae* mycelia using TRIzol reagent (Invitrogen). We used 2 µg of each total RNA sample to synthesize cDNA using a Superscript III First-Strand Synthesis SuperMix Kit (Invitrogen). The qRT-PCRs were run on a 7500 Real-Time System (Applied Biosystems) following the manufacturer’s protocols. Relative gene expression levels were calculated using the comparative 2^−ΔΔ*C*T^ method (Livak and Schmittgen, 2001). The primers used are listed in Supplementary Table S1.

Defense suppression tests were performed as previously reported (Chen *et al.*, 2015). Briefly, 10-day-old seedlings of *N. benthamiana* were treated with 1 µM flg22 (Sangon Biotech, China). The expression of the PTI-associated genes *NbACRE31*, *NbGRAS2*, and *NbPTI5* were determined by qRT-PCR. In addition, qRT-PCR was also used to determine the expression levels of the defense-related genes in the SA and jasmonic acid (JA) signaling pathways, including *PATHOGENESIS RELATED PROTEIN 1* (*NbPR1*) and *LINOLEATE 9S-LIPOXYGENASE 5* (*NbLOX*), which are specifically induced by SA signaling. The *NbEF1α* and *NbTUBULIN6* genes were used as internal controls.

### Yeast secretion assays

To validate the secretion function of the predicted SP of LtCre1 (Jacobs *et al.*, 1997; Fang *et al.*, 2016), the predicted SP coding sequence was amplified and fused in-frame into the yeast secretion-defective invertase gene in the vector pSUC2. The constructed plasmids (*pSUC2::LtCre1*^*SP*^) were transformed into the invertase-deficient yeast strain YTK12 using a Frozen-EZ Yeast Transformation II Kit (Zymo Research, Irvine, CA, USA) and screened on CMD-W media (0.67% yeast N base without amino acids, 0.075% tryptophan dropout supplement, 0.1% glucose, and 2% sucrose). Secretory function was verified by incubating the yeast transformants on YPRAA plates (1% yeast extract, 2% peptone, 2% raffinose, and antimycin A at 2 µg l^−1^) at 30 °C for 3−5 d. The recombinant YTK12 strains carrying the *pSUC2::Avr1b*^*SP*^ and *pSUC2::Mg87*^*SP*^ vectors were used as the positive and negative controls, respectively.

### ROS burst assays

Assays to measure ROS production were performed as described previously (Wang *et al.*, 2018). In brief, discs taken from the middle point of leaves of 4-week-old *N. benthamiana* plants were incubated overnight in water in a 96-well plate. The discs were then floated in inducing buffer containing 1 μM flg22, 20 nM luminol, and 20 μg ml horseradish peroxidase (HRP; Sigma-Aldrich). Luminescence was measured continuously for 30 min with a FLX800 microplate reader (BioTek).

### Agro-infiltration assays in *N. benthamiana*

To determine transient expression in *N. benthamiana*, the recombinant plasmids were transformed into *A. tumefaciens* strain GV3101 using the freeze–thaw method. The strain was cultured at 28 °C with shaking at 180 rpm overnight, after which the bacterial cells were collected, washed twice with 10 mM MgCl_2_, resuspended in infiltration buffer (10 mM MES, pH 5.7, 10 mM MgCl_2_, and 150 μM Acetosyringone), and infiltrated into 4-week-old *N. benthamiana* leaves using 1-ml needleless syringes.

For the inhibition of mammalian BAX protein-triggered cell death *in planta* (Dou *et al.*, 2008; Wang *et al.*, 2011; Chen *et al.*, 2018), the CDS of *LtCre1* without the SP was cloned into the PVX vector pGR107 (Chen *et al.*, 2015) using the specific primers CreB-SmF and CreB-SIR to construct the vector pGR-LtCre1^ΔSP^. *Agrobacterium tumefaciens* containing pGR-LtCre1^ΔSP^ was then transiently expressed in the leaves of *N. benthamiana*. Subsequently, the BAX-containing plasmid was agro-infiltrated at the same position 24 h later. Cell death symptoms were recorded ~4–5 d after the last infiltration.

The CDS of *LtCre1* without the SP was subcloned into the pEDV vector (Zhang *et al.*, 2014) using the specific primers CreSI-F and CreBI-R to construct the vector pEDV-LtCre1^ΔSP^. The pEDV-LtCre1^ΔSP^ construct was then transformed into *Burkholderia glumae* by electroporation. Leaves from 4-week-old *N. benthamiana* plants were infiltrated with *B. glumae* carrying the pEDV-LtCre1^ΔSP^ construct, and cell death symptoms were imaged ~3–4 d after infiltration.

### Yeast two- and three-hybrid assays

Several plasmids were constructed for yeast two-hybrid (Y2H) assays. The *LtCre1* CDS without the SP sequence was cloned into the *Eco*R I and *Sal* I sites of the vector pGBKT7 (Clontech) to form the BD-LtCre1^ΔSP^ plasmid, as well as into the *Eco*R I and *Xho* I sites of the pGADT7 vector (Clontech) to form the AD-LtCre1^ΔSP^ plasmid. VvRHIP1 (VIT_06s0004g00100) was amplified from the cDNA of *V. vinifera* var. ‘Thompson seedless’ using the specific primers RHIP1Y-EF and RHIP1Y-XR (Supplementary Table S1). This fragment was recombined into the pGBKT7 and pGADT7 vectors to form BD-VvRHIP1 and AD-VvRHIP1, respectively. The predicted intracellular domain of VvRGS1 (254−466 aa, VIT_01s0010g02160) was cloned into the pGBKT7 vector to form BD-VvRGS_254−466_. The Y2H assays were carried out using a GAL4-based Matchmaker Gold Yeast Two-Hybrid System (Clontech), following the manufacturer’s instructions. The constructs were co-transformed into the yeast strain Y2HGold. Transformations were selected on synthetic dropout (SD)/–Trp–Leu medium, and the interactions were confirmed by transferring the positive colonies to SD/–Trp–Leu–His–Ade medium.

For yeast three-hybrid (Y3H) assays, VvRGS_254−466_ was fused with the GAL4 DNA-binding domain, and *LtCre1* was placed under the control of the Met-repressible *MET25* promoter in the pBridge plasmid (Clontech). The pBridge vector carrying only VvRGS_254−466_ was used as the control. The recombinant vectors combined with the AD-VvRHIP1 vector were then co-transformed into yeast strain AH109. The colonies were streaked onto SD/–Leu–Trp–His medium with or without Met and cultured at 30 °C for 5 d. To measure β-galactosidase activity, yeast colonies carrying each vector combination were grown overnight at 30 °C in SD/–Leu–Trp medium with or without methionine. After incubation, β-galactosidase activity was measured with a UV spectrophotometer using O-nitrophenyl β-D-galactopyranoside (Sigma-Aldrich) as a substrate.

### Bimolecular fluorescence complementation assays

For bimolecular fluorescence complementation (BiFC) assays, the CDS of *LtCre1* without the SP was amplified and fused in-frame with N-/or C-terminal YFP to produce *LtCre1*^*ΔSP*^*-nYFP* or *LtCre1*^*ΔSP*^*-cYFP*, respectively (Liu *et al.*, 2016). The full CDS of *VvRHIP1* was cloned into the same vector to generate the *VvRHIP1-nYFP* and *VvRHIP1-cYFP* constructs. The constructed plasmids were confirmed by sequencing and transformed into *A. tumefaciens* strain GV3101. The combined *Agrobacterium* strains (*LtCre1*^*ΔSP*^*-nYFP* plus *VvRHIP1-cYFP*; *LtCre1*^*ΔSP*^*-cYFP* plus *VvRHIP1-nYFP*; *VvRHIP1-cYFP* plus *nYFP*; and *LtCre1*^*ΔSP*^*-nYFP* plus *cYFP*) were infiltrated into the epidermal leaf cells of 4-week-old *N. benthamiana* plants. YFP emissions were detected 36−48 h later using a LSM710 confocal laser scanning microscope (Zeiss).

### Expression of recombinant proteins and GST pull-down assays

The CDS of *LtCre1* without the SP was inserted into the vector pMAL-c2 to create the MBP-LtCre1^ΔSP^ fusion protein, and the CDS of *VvRHIP1* was fused into the pGEX-6p-1 vector to create the GST-VvRHIP1 fusion protein. The recombinant plasmids were transformed in *Escherichia coli* strain BL21 (DE3). The MBP-LtCre1^ΔSP^ and GST-VvRHIP1 proteins were affinity-purified using amylase resin (NEB) and a Glutathione Sepharose 4B matrix (GE Healthcare), respectively. For the pull-down assays, the MBP-LtCre^ΔSP^ protein was incubated with pre-rinsed glutathione-Sepharose 4B beads and either the GST or the GST-VvRHIP1 protein for 4 h at 4 °C with gentle shaking. After incubation, the beads were washed five times with pre-cooled immunoprecipitation (IP) buffer [50 mM Tris-HCl, pH 7.4, 150 mM NaCl, 1 mM EDTA, and 0.1% (v/v) Triton X-100], resuspended in 1× SDS-PAGE loading buffer, and boiled at 100 °C for 10 min for immunoblot analysis.

For the competitive GST pull-down assays, the VvRGS_254−466_ fragment was subcloned into the pET-32a vector to generate the His-VvRGS_254−466_ fusion protein. The recombinant His-VvRGS_254−466_ protein was expressed in *E. coli* BL21 (DE3) and then purified with Ni-nitrilotriacetic acid agarose. We incubated 1 µg of GST-VvRHIP1 or 1 µg of His-VvRGS_254−466_ with increasing amounts of MBP-LtCre1^ΔSP^ and glutathione-Sepharose 4B beads for 4 h at 4 °C with gentle shaking. After incubation, the beads were prepared for immunoblot analysis as described above.

### Co-immunoprecipitation assays

The recombinant vector Cam35S:LtCre1^ΔSP^-GFP was used for the CoIP assays. The CDS of *VvRHIP1* was amplified and fused with sequences encoding the myc epitope tag driven by the *35S* promoter at the C-terminus to generate the VvRHIP1-myc protein. Both constructs were transiently expressed in *N. benthamiana* leaves as described above. At 48 h after agro-infiltration, total proteins were extracted from the leaves using protein extraction buffer (50 mM Tris-HCl, pH 7.4, 150 mM NaCl, 1 mM EDTA, 1% Triton X-100). The proteins were incubated at 4 °C for 4 h with GFP-Trap agarose beads (Sigma-Aldrich). The immuno-precipitated proteins were immunoblotted with HRP-conjugated anti-myc antibody (Roche) or anti-GFP antibodies (Sigma-Aldrich). The proteins were then immunoblotted with anti-mouse HRP-conjugated secondary antibody (CWBio, Beijing, China).

### Split-luciferase complementation assays

The split-luciferase complementation assays were performed as previously described (Yang *et al.*, 2019). In brief, the CDS of *LtCre1* without the SP was cloned in-frame with C-terminal LUC (cLUC) to generate the LtCre1^ΔSP^-cLUC construct, and the CDS of *VvRHIP1* was cloned in-frame with N-terminal LUC (nLUC) to generate the VvRHIP1-nLUC construct. Cells of *A. tumefaciens* strain GV3101 containing the desired nLUC or cLUC plasmids were mixed and infiltrated into the leaf cells of *N. benthamiana* plants. At 48 h after infiltration, the leaves were collected and incubated with 0.5 µM luciferin in the dark for 5 min. Relative luciferase activity was captured using a Lumazone PyLoN 2048B cooled charge-coupled device imaging apparatus (Roper Scientific, Acton, MA, USA).

### Sugar starvation treatment

The sugar starvation treatment was performed as described previously (Huang *et al*, 2015). Ten-day-old *N. benthamiana* seedlings were transferred to the dark for 2 d, and then sprayed with half-strength MS medium supplemented with either 0% or 3% D-glucose and left for 3 h under constant low light.

### Transcriptome analysis

Total RNA was isolated from *N. benthamiana* leaves using a *TransZol* Plant RNA kit (TransGen Biotech). Wild-type and *LtCre1*-overexpressing seedlings (two biological replicates each) were grown under a 16/8-h light/dark cycle (130 µmol m^–2^ s^–1^) at 26 °C in a greenhouse. RNA samples were sequenced using an Illumina HiSeq platform. The RNA-seq data were aligned to the *N. benthamiana* reference genome (v1.0.1) using Hisat2 v2.0.4 (Kim *et al*, 2015) with default parameters. Unique mapped reads were used for subsequent analysis. Differentially expressed genes were identified using the DEGSeq R package (1.12.0) with the criteria of adjusted *P*-value (qvalue) <0.005 and fold-change >2. Gene Ontology (GO) enrichment analysis was performed using the GOseq R package (Young *et al*, 2010), and Kyoto Encyclopedia of Genes and Genomes (KEGG) enrichment analysis was performed using the KOBAS software (Mao *et al*, 2005) with default parameters. All RNA-seq data produced in this study have been deposited in the NCBI GEO database under accession number GSE189162.

### Statistical analysis

Significant differences were analysed using one-way ANOVA followed by Duncan’s multiple range test in the IBM SPSS Statistics software.

## Results

### LtCre1 is required for the full virulence of *L. theobromae*

**Fig. 1.**
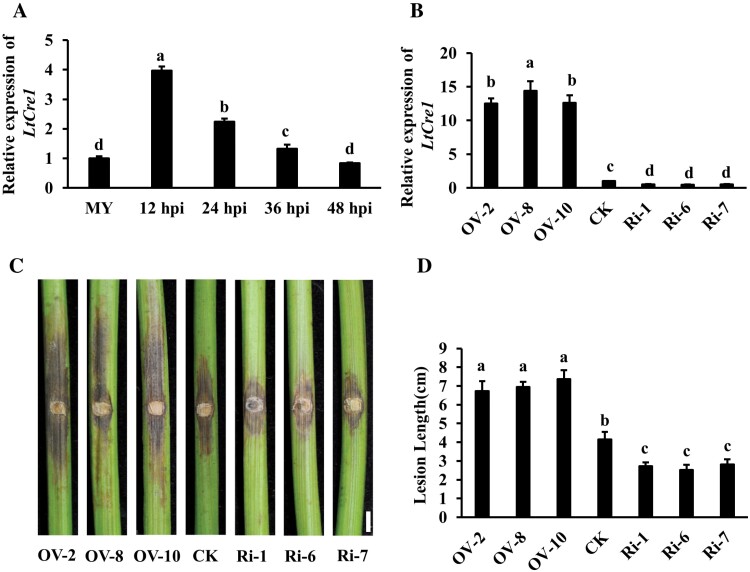
LtCre1 contributes to *Lasiodiplodia theobromae* virulence. (A) Relative expression pattern of the effector gene *LtCre1* during *L. theobromae* infection as determined by quantitative reverse-transcription (qRT-)PCR. The grape cultivar ‘Summer Black’ was inoculated with *L. theobromae* and samples of stem phloem were collected at 12–48 h post-inoculation (hpi). The fungal *Actin* gene was used as the internal reference for transcript normalization. MY, *L. theobromae* mycelia grown in potato dextrose agar (PDA) plates for 3 d. Expression is relative to that of MY, the value of which was set as 1. (B) Relative expression levels of *LtCre1* in *L. theobromae* transformants overexpressing *LtCre1* (OV-2, -8, and -10) or transformants with RNAi-knockdown of *LtCre1* (RI-1, -6, and -7). The transformants were grown on PDA plates. The *Actin* gene of *L. theobromae* was used as the internal reference gene for transcript normalization. Expression is relative to that in the wild-type *L. theobromae* strain CSS-01s control (CK), the value of which was set as 1. (C) Detached shoots of ‘Summer Black’ grape 6 d after inoculation with the *LtCre1*-overexpression and RNAi transformants, compared with the control strain CSS-01s. Scale bar is 1 cm. (D) Mean lesion lengths on the shoots shown in (C). All data are means (±SD) of three biological replicates. Different letters indicate significant differences among means as determined using one-way ANOVA followed by Duncan’s multiple range test (*P*<0.05).

Compared to control mycelia grown on potato dextrose agar plates, expression of *LtCre1* was significantly up-regulated in *L. theobromae* isolate CSS-01s during the early stages of infection of the stems of the grape cultivar ‘Summer Black’ (12−36 h post-inoculation, hpi; Fig. 1A; Supplementary Fig. S1A), with the level of transcripts peaking at 12 hpi and then gradually declining to control levels by 48 hpi. This suggested that *LtCre1* might play a role in *L. theobromae* infection.

To investigate whether LtCre1 was required for *L. theobromae* virulence, stable *LtCre1-*overexpression transformants and stable RNAi-mediated *LtCre1*-knockdown transformants were generated using the PEG-mediated protoplast transformation method. The morphology of the colonies and mycelial growth rates of the transformants did not differ significantly from those of the WT strain (Supplementary Fig. S1B, C). Subsequent expression analyses showed that, compared to the WT control, *LtCre1* was significantly up-regulated in the overexpressing transformants (10-fold increase in the expression level) and significantly down-regulated in the *LtCre1*-RNAi transformants (2-fold decrease in the expression level; Fig. 1B; Supplementary Fig. S1D). ‘Summer Black’ grape stems inoculated with the *LtCre1*-overexpressing transformant exhibited markedly longer lesions than stems inoculated with the *L. theobromae* WT; conversely, stems inoculated with the *LtCre1*-RNAi transformants exhibited markedly shorter lesions than stems inoculated with the WT (Fig. 1C, D). Taken together, these results indicated that *LtCre1* could play important roles in the pathogenicity of *L. theobromae*.

### Ectopic expression of *LtCre1* increases the susceptibility of *N. benthamiana* to *L. theobromae*

**Fig. 2.**
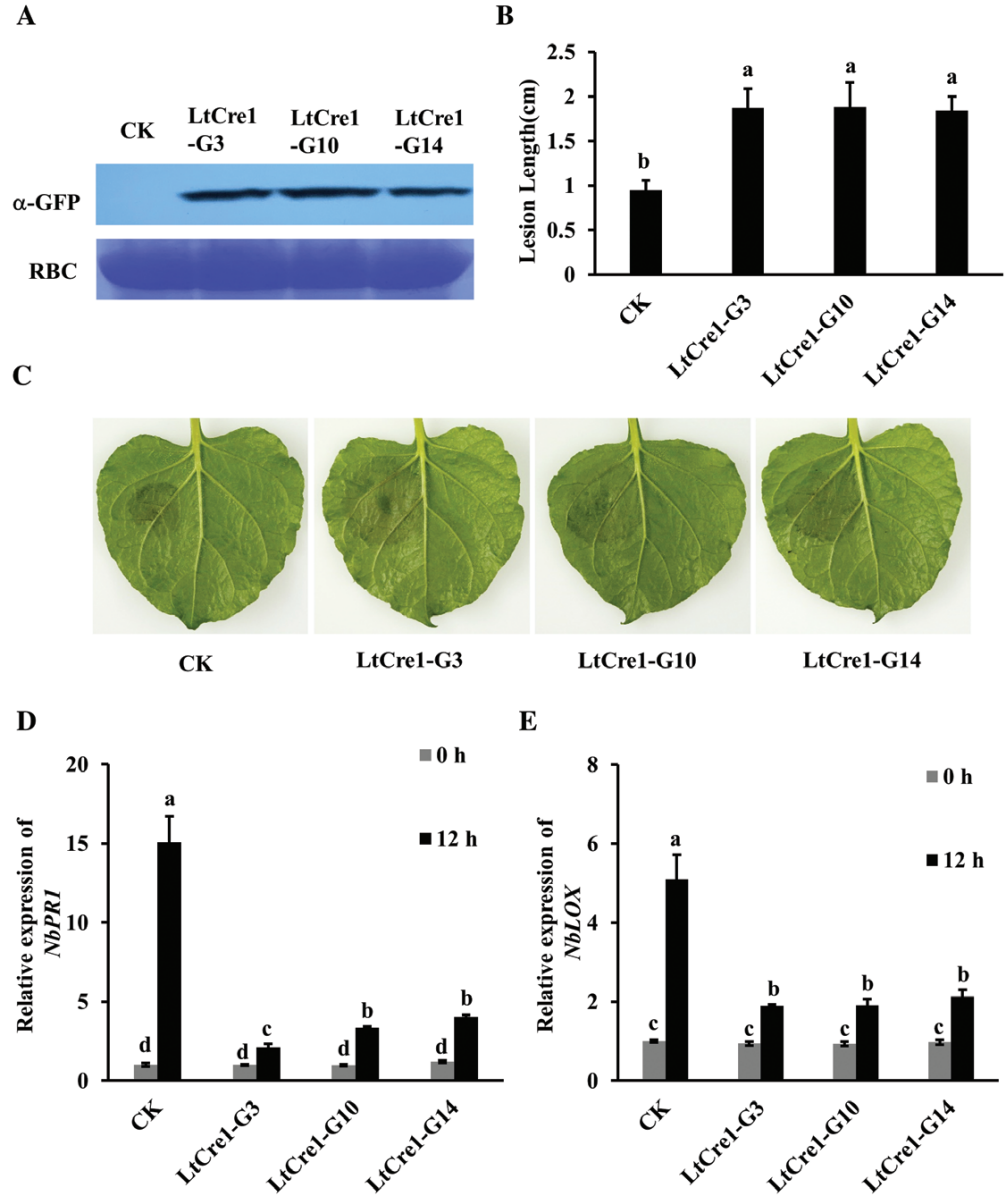
Heterologous expression of *LtCre1* increases the susceptibility of *Nicotiana benthamiana* to *Lasiodiplodia theobromae*. (A) Western blots using an anti-GFP monoclonal antibody showing the expression of *LtCre1-GFP* in the leaves of the wild-type (CK) and three lines of transgenic *N. benthamiana* overexpressing *LtCre1* (T3 generation). Rubisco (RBC) stained with Coomassie G250 was used as the loading control. (B) Mean diameters of lesions induced by *L. theobromae* strain CSS-01s on the leaves of the wild-type and the *LtCre1*-overexpressing lines (36 h after inoculation). Data are means (±SD) of 10 independent biological replicates. (C) Representative images of leaves of the control and transgenic lines at 36 h after *L. theobromae* inoculation. (D, E) Relative expression levels of the defense-related genes (D) *NbPR1* and (E) *NbLOX* in the control and *N. benthamiana LtCre1*-overexpressing lines at 0 h and 12 h after inoculation with *L. theobromae*, as determined by qRT-PCR using *NbEF1α* as the internal reference. Expression is relative to that in the control at 0 h, the value of which was set as 1. For results using *NbTUBLIN6* as the reference see Supplementary Fig. S3. Data are means (±SD) of three independent biological replicates. Different letters indicate significant differences among means as determined using one-way ANOVA followed by Duncan’s multiple range test (*P*<0.05).

To further characterize the function of LtCre1, we generated several independent transgenic *N. benthamiana* lines harboring the *CaMV35S::LtCre1-GFP* construct, 20 of which exhibited high levels of *LtCre1* expression. Three of these lines were selected for further study (LtCre1-GFP3, LtCre1-GFP10, and LtCre1-GFP14; Supplementary Fig. S2). Western blots showed that LtCre1-GFP was properly expressed at the expected size in the T3 generation (Fig. 2A). There were no visible morphological differences between transgenic *N. benthamiana* in the T3 generation and the WT control, indicating that LtCre1 had an insignificant effect on tobacco plant growth (Supplementary Fig. S2C–E).

After inoculation with *L. theobromae* CSS-01s, lesions on the detached leaves of transgenic *N. benthamiana* overexpressing *LtCre1* were significantly larger than those on the detached leaves of the WT control (Fig. 2B, C). At 12 h after *L. theobromae* inoculation, the defense-related genes *NbPR1* and *NbLOX* were significantly suppressed in the T3 transgenic *N. benthamiana* plants overexpressing *LtCre1* compared with the control plants (Fig. 2D; Supplementary Fig. S3). Furthermore, the expression levels of the PTI-associated genes *NbACRE31*, *NbGRAS2* and *NbPTI5* in response to treatment with flg22 were significantly decreased in the plants overexpressing *LtCre1* compared with the controls (Supplementary Fig. S4). Collectively, these results indicated that LtCre1 might suppress the basal immunity of *N. benthamiana*.

### LtCre1 suppresses BAX-triggered cell death

**Fig. 3.**
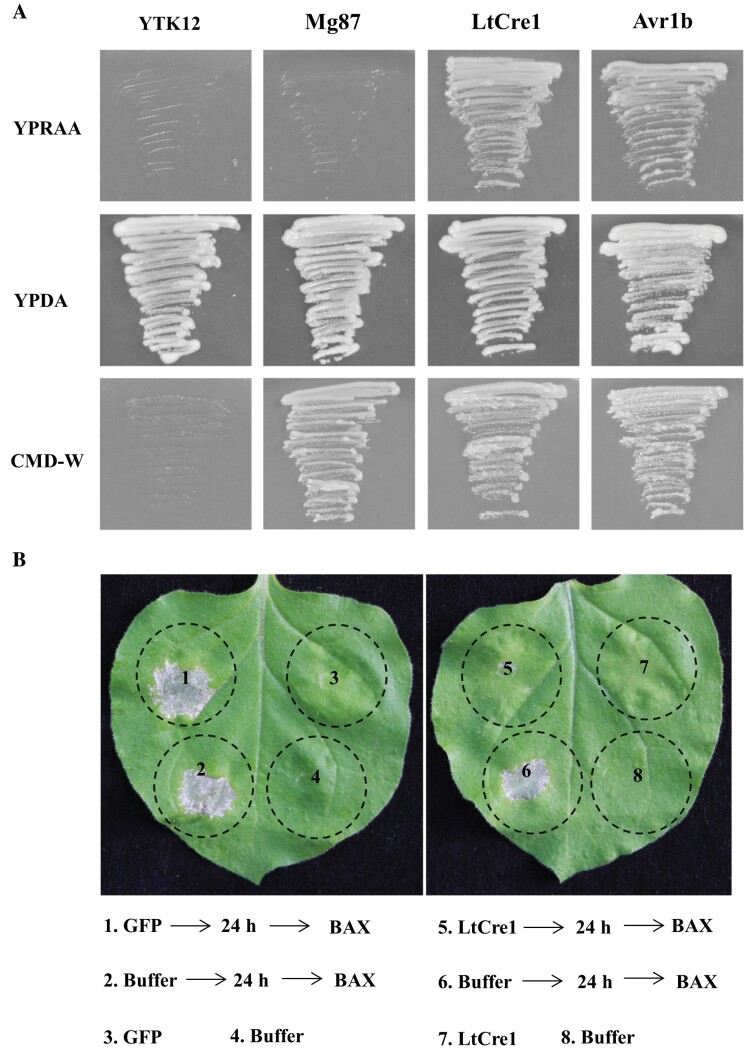
LtCre1 suppresses BAX-triggered cell death in *Nicotiana benthamiana*. (A) Functional validation of the putative signal peptides of LtCre1 using yeast invertase secretion assays. The yeast strain YTK12 transformed with the signal peptide sequence of LtCre1 and fused in-frame with the invertase gene SUC2 was grown on YPRAA medium (1% yeast extract, 2% peptone, 2% raffinose, and antimycin A at 2 µg l^−1^) with raffinose as the sole carbon source. Positive transformants were selected on CMD-W medium (0.67% yeast N base without amino acids, 0.075% tryptophan dropout supplement, 0.1% glucose, and 2% sucrose). The untransformed yeast strains did not grow on either YPRAA or CMD-W media, whereas yeast growth and viability was equivalent across strains on YPDA medium (1% yeast extract, 2% peptone, 2% glucose, and 0.02% adenine hemisulfate). The signal peptide Avr1b from *Phytophthora sojae* was used as the positive control, while the N-terminal sequence of Mg87 from *Magnaporthe oryzae* was used as the negative control. (B) Representative images showing that transiently expressed LtCre1^ΔSP^ (LtCre1 lacking the signal peptide) suppressed BAX-triggered cell death in leaves of 4-week-old *N. benthamiana* plants.

The LtCre1 protein contains a putative N-terminal secretory SP, (aa 1−17), an RXLG-EER motif starting at position 49, and a zinc-finger domain at the C-terminus. The mature LtCre1 polypeptide contains 331 amino acids, including 10 cysteine residues, and has a molecular mass of 36.1 kD (Supplementary Fig. S5A). To test the functionality of the predicted SP of LtCre1, we used a yeast secretion system to measure the invertase activity (Jacobs *et al.*, 1997; Fang *et al.*, 2016). We found that yeast strain YTK12 carrying the LtCre1 SP grew on YPRAA medium with raffinose as the sole carbon source, similar to the positive control pSUC2-Avr1b (Fig. 3A), confirming that the LtCre1 SP functions correctly to support invertase secretion in yeast.

Subcellular localization analysis using the agro-infiltration-mediated transient expression of *CaMV35S::LtCre1*^*ΔSP*^*-GFP* and *CaMV35S::GFP* in *N. benthamiana* epidermal leaf cells showed that the LtCre1^ΔSP^-GFP protein (i.e. LtCre1 lacking the signal peptide) was largely restricted to the nucleus and the cytoplasm, unlike the GFP control (Supplementary Fig. S5B).

In *N. benthamiana* leaves transiently expressing *LtCre1*^*ΔSP*^, BAX-triggered cell death was strongly inhibited; in contrast, BAX-induced necrosis was unaffected in control *N. benthamiana* leaves agro-infiltrated only with the GFP protein or buffer (Fig. 3B). Gene expression analysis verified that *LtCre1*, *BAX*, and *GFP* were appropriately expressed after their respective infiltrations (Supplementary Fig. S6). In previous studies, the *Burkholderia glumae*-pEDV system has been employed to identify putative effectors that suppress HR in *N. benthamiana* (Zhang *et al.*, 2014). We found that LtCre1^ΔSP^ also suppressed *B. gluma*e-triggered cell death (Supplementary Fig. S7). These results suggested that LtCre1 plays a role in the plant immune response.

### LtCre1 interacts with the grapevine protein VvRHIP1

**Fig. 4.**
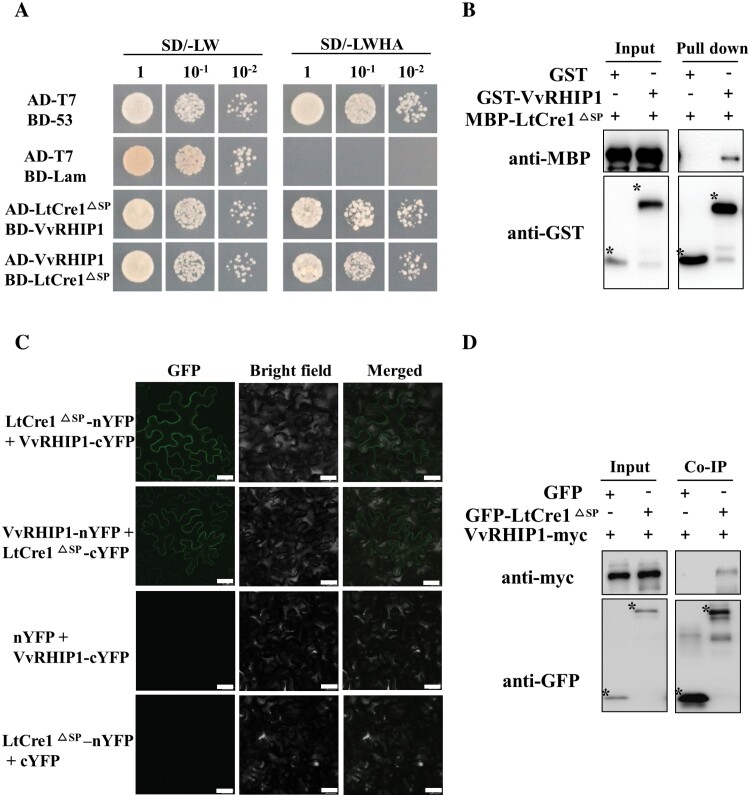
LtCre1 physically interacts with VvRHIP1. (A) Yeast two-hybrid assays demonstrating the interaction between LtCre1 and VvRHIP1. Serial dilutions of yeast transformants were cultured on synthetic double-dropout medium either lacking leucine and tryptophan (SD/–LW) or quadruple-dropout medium lacking leucine, tryptophan, histidine, and adenine (SD/–LWHA). The plates were imaged 5 d after inoculation. Yeast cells harboring AD-T7 paired with either BD-53 or BD-Lam vectors were used as the positive and negative controls, respectively. LtCre1^ΔSP^, LtCre1 lacking the signal peptide. (B) *In vitro* pull-down assays of maltose binding protein (MBP)-LtCre1^ΔSP^ by GST-VvRHIP1. The GST pull-down assays used recombinant MBP-LtCre1^ΔSP^ and GST-VvRHIP1 fusion proteins produced in *E. coli*. GST-tagged and MBP-LtCre1^ΔSP^ fusion proteins were used as the negative controls. Interacting proteins were identified by immunoblotting using anti-MBP antibodies or anti-GST antibodies before (5% input) and after pull-down. (C) Bimolecular fluorescence complementation assay showing the interaction between LtCre1 and VvRHIP1. LtCre1^ΔSP^ and VvRHIP1 were fused in-frame with the YFP N-terminal region (nYFP) or C-terminal region (cYFP) and agro-infiltrated into *Nicotiana benthamiana* epidermal leaf cells. Leaves co-expressing VvRHIP1-cYFP plus nYFP or LtCre1^ΔSP^ -nYFP plus cYFP were used as negative controls. Confocal images were captured 48 h after infiltration. Bright field images show differential interference contrast (DIC) and merged images show GFP and DIC images combined. Scale bars are 25 µm. (D) Co-immunoprecipitation (Co-IP) of LtCre1 and VvRHIP1. GFP-LtCre1^ΔSP^ or the GFP protein was transiently co-expressed with VvRHIP1-myc in *N. benthamiana* leaf epidermal cells. Co-IP was performed using GFP-Trap agarose beads, and the isolated protein was immunoblotted with anti-myc antibodies to detect VvRHIP1-myc.

To identify the proteins interacting with LtCre1, *LtCre1*^*ΔSP*^ was used as bait to screen a cDNA library derived from grapevine leaves infected with *L. theobromae* CSS-01s. This analysis identified 15 independent clones corresponding to the LtCre1-interacting proteins (CIPs). A single copy of the gene encoding CIP12, which strongly interacted with LtCre1^ΔSP^, was located on *V. vinifera* chromosome 6 (VIT_06s0004g00100). CIP12 was homologous to Arabidopsis RGS1-HXK1-INTERACTING PROTEIN 1 (AtRHIP1, AT4G26410), and we therefore named this gene *VvRHIP1*. The VvRHIP1 protein had the highest sequence identity with NbRHIP1 (62.7%), followed by AtRHIP1 (60.8%) (Supplementary Fig. S8). Y2H assays demonstrated that co-transformed yeast cells grew on the ­selection medium lacking Leu, Trp, His, and Ade, confirming the interaction between full-length VvRHIP1 and LtCre1^ΔSP^ in yeast (Fig. 4A).

Consistent with this, *in vitro* pull-down assays using *E. coli* cells expressing MBP-tagged LtCre1^ΔSP^ and GST-VvRHIP1 showed that the MBP-LtCre1^ΔSP^ protein was effectively pulled down by the GST-tagged VvRHIP1 but not by the GST control (Fig. 4B). These results suggested that LtCre1^ΔSP^ directly interacted with VvRHIP1.

We examined the subcellular localization of the LtCre1–VvRHIP1 interaction by transiently co-expressing plasmids containing *LtCre1*^*ΔSP*^*-nYFP*/*VvRHIP1-cYFP* and *VvRHIP1-nYFP*/*LtCre1*^*ΔSP*^*-cYFP* in *N. benthamiana* leaf epidermal cells via agro-infiltration. The surfaces of cells infiltrated with ­plasmids containing *LtCre1*^*ΔSP*^ and *VvRHIP1* exhibited strong fluorescence signals, while cells infiltrated with control plasmids (*nYFP*/*VvRHIP1-cYFP* and *LtCre1*^*ΔSP*^*-nYFP*/*cYFP*) did not (Fig. 4C).

The interaction between LtCre1 and VvRHIP1 was further demonstrated using CoIP in which recombinant GFP-tagged LtCre1^ΔSP^ and VvRHIP1-myc proteins were transiently co-expressed in *N. benthamiana* leaf epidermal cells through agro-infiltration. Immunoblotting showed that VvRHIP1-myc, but not the GFP control, was co-immunoprecipitated by GFP-Trap agarose beads (Fig. 4D); the anti-myc antibody was also precipitated in the presence of GFP-LtCre1^ΔSP^, but not in the presence of the GFP control. Finally, we used a split-luciferase assay to validate the interaction between LtCre1 and VvRHIP1. Strong luciferase activity was detected when VvRHIP1-nLUC was transiently co-expressed with ­LtCre1-cLUC in *N. ­benthamiana* cells, whereas no luciferase signal was observed in the negative controls (Supplementary Fig. S9). Together, these results demonstrated that LtCre1 physically interacted with VvRHIP1 both *in vitro* and *in vivo*.

### VvRHIP1 positively regulates plant immunity to *L. theobromae*

**Fig. 5.**
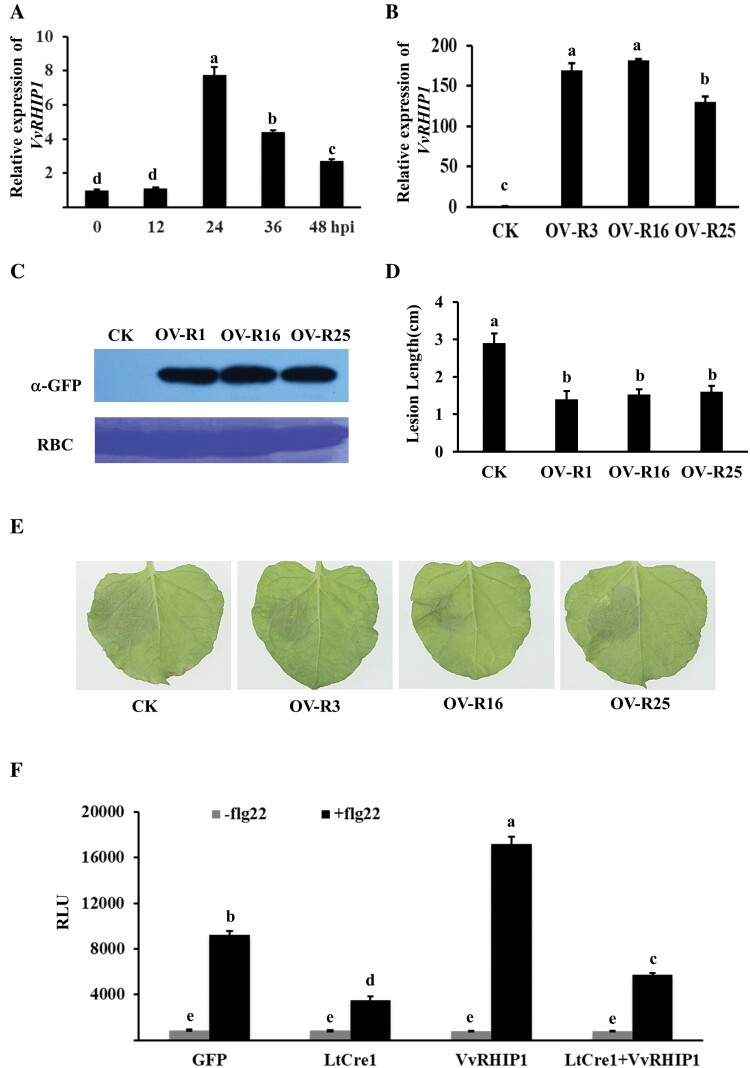
VvRHIP1 positively regulates plant immunity. (A) Relative expression of *VvRHIP1* in stem phloem samples of ‘Summer Black’ grapevine during *Lasiodiplodia theobromae* infection, as determined using qRT-PCR. *VvEF1γ* was used as the internal reference, and expression is relative to that at 0 h, the value of which was set as 1. (B) Relative expression of *VvRHIP1* in transgenic *Nicotiana benthamiana* lines overexpressing *VvRHIP1*. *NbEF1α* was used as the internal reference for normalization. CK, wild-type control. (C)Western blots showing VvRHIP1 fused with the GFP tag in the *N. benthamiana* lines overexpressing *VvRHIP1*. Rubisco (RBC) proteins stained with Coomassie G250 were used as the loading control. (D) Lesion diameters in the *N. benthamiana* control and *VvRHIP1*-overexpressing lines at 72 h after inoculation with *L. theobromae*. Data in (A, B, D) are means (±SD) of 10 independent biological replicates. (E) Typical leaf phenotypes of the *N. benthamiana* control and *VvRHIP1*-overexpressing lines at 72 h after inoculation with *L. theobromae*. (F) ROS production elicited by flg22. Relative luminescence units (RLU) reflect the relative amount of H_2_O_2_ accumulated in *N. benthamiana* leaf strips treated with 1 µM flg22. Data are means (±SD) of three independent experiments. In all graphs, different letters indicate significant differences among means as determined using one-way ANOVA followed by Duncan’s multiple range test (*P*<0.05).

To investigate the molecular role of VvRHIP1 in plant immunity, we first examined expression patterns in stem phloem of ‘Summer Black’ grapevine in response to *L. theobromae* infection and found that *VvRHIP1* was up-regulated at 24−48 hpi, with a peak at 24 hpi (Fig. 5A; Supplementary Fig. S10A). We then introduced *35S::VvRHIP1-GFP* into *N. benthamiana* to obtain overexpression lines (OV-R3, OV-R16, and OV-R25; Fig. 5B, C; Supplementary Fig. S10B). Upon challenge by *L. theobromae*, the transgenic *N. benthamiana* overexpressing *VvRHIP1* had significantly shorter lesions than the WT controls (Fig. 5D, E). In addition, the defense-marker genes *NbPR1* and *NbLOX* were significantly up-regulated in the transgenic plants compared to the WT control (Supplementary Fig. S10C–F).

Next, we investigated whether *VvRHIP1* affected basal defense responses by determining ROS production in *N. benthamiana* leaves expressing *GFP*, *LtCre1*^*ΔSP*^*-GFP*, *VvRHIP1-HA*, or *LtCre1*^*ΔSP*^*-GFP+VvRHIP1-HA*. We found that flg22-induced ROS bursts were significantly greater in the presence of *VvRHIP1* as compared to the *GFP* control, and that the expression of *LtCre1*^*ΔSP*^ suppressed *VvRHIP1*-induced ROS production (Fig. 5F). Finally, PTI-associated genes were up-regulated in *N. benthamiana* overexpressing *VvRHIP1* in response to flg22 treatment (Supplementary Fig. S11). Together, these results indicated that VvRHIP1 acts as a positive regulator of plant immunity.

### LtCre1 disrupts the VvRHIP1–VvRGS1 complex

**Fig. 6.**
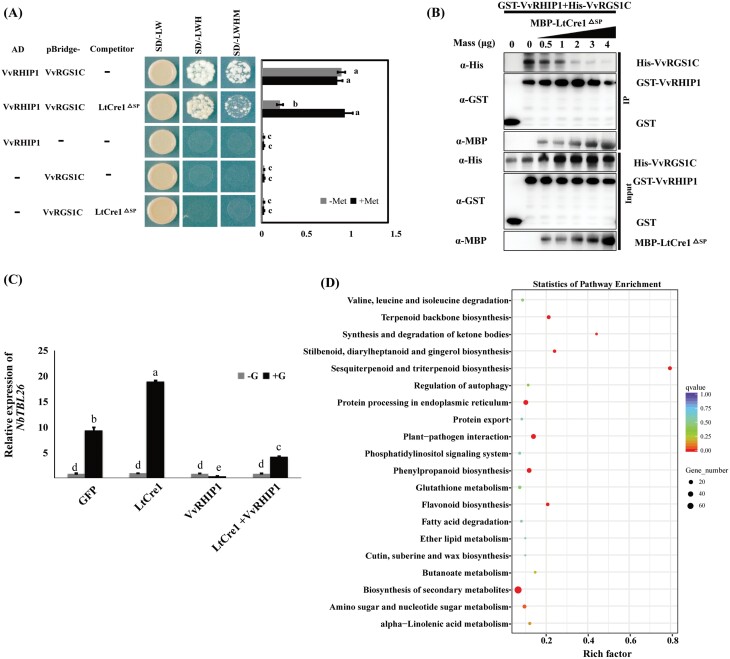
LtCre1 disrupts the association between VvRHIP1 and VvRGS1. (A) Yeast three-hybrid assays showing the inhibition of the interaction between VvRHIP1 and VvRGS1 by LtCre1 in yeast cells. VvRHIP1 was cloned and fused in -frame with the GAL4-activation domain (AD-VvRHIP1). VvRGS1C was fused into the GAL4-binding domain, and LtCre1^ΔSP^ (LtCre1 lacking the signal peptide) was expressed in the pBridge plasmid driven by the Met-repressible pMET25 promoter. Plasmids containing VvRHIP1, VvRGS1C, or VvRGS1C paired with LtCre1^ΔSP^ were used as negative controls. Yeast cells were imaged after 5 d of culture on SD medium lacking Leu and Trp (SD/–LW), on SD medium lacking Leu, Trp, and His (SD/–LWH), and on SD medium lacking Leu, Trp, His, and Met (SD/–LWHM). To measure β-galactosidase activity, three independent clones of each combination were cultured in SD liquid medium lacking Leu and Trp with or without methionine (+Met and –Met, respectively) at 30 °C to OD_600_=2.0. The β-galactosidase activity was determined using O-nitrophenyl-*β* -D-galactopyranoside as a substrate. Data are means (±SD) of at least three independent experiments. Different letters indicate significant differences among means as determined using one-way ANOVA followed by Duncan’s multiple range test (*P*<0.05). (B) LtCre1 interferes with the interaction between VvRHIP1 with VvRGS1 *in vitro*. The purified GST-VvRHIP1 protein was co- precipitated with His-VvRGS1C using glutathione-Sepharose 4B beads beads with or without the MBP-LtCre1^ΔSP^ protein. Immunoblotting was then performed with the antibodies indicated. (C) Relative expression of *NbTBL26* as determined by qRT-PCR in the leaves of transgenic *Nicotiana benthamiana* overexpressing *GFP*, *LtCre1-GFP*, *VvRHIP1-HA*, or *LtCre1-GFP*+*VvRHIP1-HA* in plants treated with half-strength MS medium supplemented with either 0% D-glucose (–G) or 3% D-glucose (+G) for 3 h. Expression levels were normalized to *NbEF1α*. Data are means (±SD) of three independently replicated experiments. Different letters indicate significant differences among means as determined using one-way ANOVA followed by Duncan’s multiple range test (*P*<0.05). (D) KEGG pathway enrichment of the differentially expressed genes (DEGs) between the transgenic *N. benthamiana* plants overexpressing *LtCre1* and the wild-type. The size of each dot reflects the total number of DEGs associated with the corresponding pathway.

AtRHIP1 has been shown to serve as a physical scaffold for AtRGS1 and for AtHXK1, which are both glycolysis-independent glucose-signaling sensors (Moore *et al.*, 2003; Chen and Jones, 2004; Urano *et al.*, 2012; Fu *et al.*, 2014; Huang *et al.*, 2015). Sequence alignment of amino acids revealed that VvRGS1 shares 62% identity with AtRGS1 (Supplementary Fig. S12), and that the sequence of VvRHIP1 displays 60% shared identity with AtRHIP1 (Supplementary Fig. S8). The interaction between LtCre1 and VvRHIP1 prompted us to investigate whether LtCre1 interfered with the association between VvRHIP1 and the *V. vinifera* RGS1 homolog. First, we cloned the intracellular domain of VvRGS1 (hereafter abbreviated to VvRGS1C) and used Y2H assays to confirm that VvRGS1C interacted with VvRHIP1 but not with LtCre1^ΔSP^ (Supplementary Fig. S13). Next, Y3H assays were performed to assess the competition between LtCre1 and VvRHIP1 in binding with VvRGS1, using *LtCre1*^*ΔSP*^ driven by a Met-suppressible promoter. In the absence of Met, LtCre1^ΔSP^ significantly decreased the interaction between VvRHIP1 and VvRGS1C (Fig. 6A), and pull-down assays indicated that LtCre1^ΔSP^ inhibited this interaction in a dose-dependent manner (Fig. 6B). Together, these results demonstrated that the effector LtCre1 competes with VvRHIP1 to bind to VvRGS1, thereby disrupting the VvRHIP1–VvRGS1 complex.

### LtCre1 participates in regulating the expression of genes associated with plant sugar signaling

It has previously been shown that both RGS1 and HXK1 regulate the expression of *TBL26*, which encodes a protein involved in the synthesis and deposition of secondary wall cellulose (Cho *et al.*, 2006; Grigston *et al.*, 2008; Urano *et al.*, 2012; Huang *et al.*, 2015). In addition, glucose-induced *TBL26* expression is regulated by RHIP1 (Huang *et al.*, 2015). Therefore, to investigate whether LtCre1 affected the plant glucose-signaling pathway, we expressed *LtCre1* and *VvRHIP1* in *N. benthamiana* leaves and performed a glucose starvation treatment. We found that *NbTBL26* was significantly up-regulated in transgenic *N. benthamiana* overexpressing *LtCre1*, and that it was significantly down-regulated when *VvRHIP1* was overexpressed (Fig. 6C). In addition, *VvRHIP1* induced significant down-regulation of *NbTBL26* in response to glucose, and this effect was significantly reversed by co-expression of *LtCre1*. Together, these results suggested that LtCre1 plays an important role in glucose-induced *TBL26* expression.

To further explore the effects of *LtCre1*-overexpression on key plant metabolic pathways in *N. benthamiana*, we performed RNA-seq analysis to identify differentially expressed genes (DEGs) between 6-week old WT and transgenic *LtCre1*-overexpressing plants under normal growth conditions. More than 40 million paired-end clean reads were obtained from each sample and ~90% of the reads were successfully mapped on to the *N. benthamiana* reference genome (Supplementary Table S2). Furthermore, ~81% of the reads were uniquely mapped to a single genomic locus. Across all sets of DEGs between the *LtCre1*-overexpressing lines and the WT, we identified 242 shared DEGs (Supplementary Table S3; Supplementary Fig. S14). We found that 10 KEGG pathways were significantly enriched in these shared DEGs (*P*≤0.05; Fig. 6D), of which the five associated with the largest numbers of DEGs were ‘biosynthesis of secondary metabolites’, ‘protein processing in endoplasmic reticulum’, ‘plant–pathogen interaction’, ‘phenylpropanoid biosynthesis’, ‘amino sugar and nucleotide sugar metabolism’, and ‘terpenoid backbone biosynthesis’. Within the ‘amino sugar and nucleotide sugar metabolism’ pathway, the DEGs encoding chitinase and glucose-4-epimerase were significantly up-regulated while DEGs encoding hexokinase were down-regulated (Supplementary Table S3; Supplementary Fig. S15). Of the DEGs associated with ‘amino sugar and nucleotide sugar metabolism’, six were independently validated using qRT-PCR (Supplementary Figs S16, S17). Together, these results indicated that LtCre1 might interfere with sugar-signaling pathways to promote *L. theobromae* infection.

## Discussion

Grapevine trunk diseases (GTDs) are destructive and as a group are currently considered to be the most prevalent threat to viticulture in all vine-producing areas (Ramsdell, 1995; Gramaje *et al.*, 2018). Phytopathogenic microbes have been reported to employ a variety of effector proteins that target different essential host compartments or pathways to suppress plant immunity and promote pathogen infection (Lo Presti *et al.*, 2015; Franceschetti *et al.*, 2017; Friesen and Faris, 2021). Hence, elucidation of the molecular mechanisms of the secreted effectors of GTD fungi helps in the identification of novel plant immunity components. In this study, we identified and characterized a novel effector, LtCre1, from *L. theobromae*. We found that *LtCre1* was strongly up-regulated in *L. theobromae* during the early stages of its infection of grapevine, with expression peaking at 12 hpi and declining thereafter (Fig. 1A). RNAi-knockdown of *LtCre1* significantly reduced *L. theobromae* virulence while stable overexpression significantly increased virulence (Fig. 1B, C). In addition, heterologous expression of *LtCre1* in *N. benthamiana* increased susceptibility to *L. theobromae* (Fig. 2). Thus, LtCre1 is essential for the full virulence of *L. theobromae*, indicating that it is a significant virulence factor.

RXLR motifs can function as translocation signals for some intracellular effectors of plant filamentous pathogens (Whisson *et al.*, 2007; Dou *et al.*, 2008; Dou and Zhou, 2012; Anderson *et al.*, 2015). Several studies have also shown that variations in amino acids in the RXLR motif can aid the entry of the effector into host cells (Kale *et al.*, 2010; Kemen *et al.*, 2011; Links *et al.*, 2011; Sharma *et al.*, 2015). In LtCre1, the canonical RXLR-EER motif is replaced by RALG-EER (Supplementary Fig. S5A). However, *Hyaloperonospora arabidopsidis* ATR5Emoy2 has been shown to harbor a canonical EER motif (without an RXLR motif) that functions similarly to the RXLR-EER motif (Bailey *et al.*, 2011), and hence future work should investigate whether the RALG-EER motif of LtCre1 also functions similarly to RXLR-EER.

As an opportunistic fungal pathogen, *L. theobromae* probably experiences a prolonged endophytic or latent phase in a living host plant (Chethana *et al.*, 2016; Paolinelli-Alfonso *et al.*, 2016). Effector proteins suppress or inhibit plant cell death and thus ensure pathogen colonization during the biotrophic phase of infection (Jones and Dangl, 2006; de Jonge *et al.*, 2011; Lo Presti *et al.*, 2015; Jones *et al.*, 2016; Zhou and Zhang, 2020). Our results demonstrated that the expression of *LtCre1* in *N. benthamiana* markedly suppressed BAX-induced cell death (Fig. 3) and *B. glumae*-induced non-host cell death (Supplementary Fig. S7), suggesting that LtCre1 might regulate plant immunity to facilitate *L. theobromae* infection. Furthermore, three PTI-associated genes (*NbACRE3*, *NbGRAS2*, and *NbPTI5*) were also significantly suppressed in *N. benthamiana* plants overexpressing *LtCre1* (Supplementary Fig. S4). Thus, our results suggested that *LtCre1*, which encodes an effector with an EER motif, functions as a suppressor of plant immunity during the biotrophic stage of *L. theobromae* infection.

To facilitate host infection, pathogen effectors target and directly hijack plant proteins associated with immune resistance (Deslandes and Rivas, 2012; Shao *et al.*, 2021). Interestingly, the *L. theobromae* candidate effector LtCre1 physically interacted with grapevine VvRHIP1 both in yeast and *in planta* (Fig. 4; Supplementary Fig. S9). To the best of our knowledge, this is the first report showing that GTD-associated fungal effectors target G protein-mediated sugar signaling. Infection by *L. theobromae* significantly induced the expression of *VvRHIP1* in grapevine (Fig. 5; Supplementary Fig. S10), indicating that it might also be involved in the response to *L. theobromae* infection. Overexpression of *VvRHIP1* reduced colonization of *N. benthamiana* leaves by *L. theobromae*, indicating that it is important for host immune responses. In addition, we observed that expression of *VvRHIP1* strongly up-regulated plant defense genes, including *NbPR1* and *NbLOX* (Supplementary Fig. S10) as well as PTI-associated genes (*NbACRE3*, *NbGRAS2*, and *NbPTI5*; Supplementary Fig. S11). Thus, we propose that VvRHIP1 acts as a positive regulator of plant resistance to *L. theobromae*.

Plant pathogens have evolved adaptive strategies to retrieve intracellular nutrients from host plants and to successfully establish infection *in planta*; in opposition, plants employ effective strategies to protect themselves against such invasion (Pieterse *et al.*, 2012). Therefore, modulations of plant secondary metabolites, including sucrose and sucrose by-products (i.e. glucose and fructose) are central to plant–pathogen interactions (Piasecka *et al.*, 2015; Pusztahelyi *et al.*, 2015). For example, the maize mutants *id1* and *sugary1*, which have an altered sugar metabolism, are less susceptible to the pathogenic fungus *Ustilago maydis* (Kretschmer *et al.*, 2017). Indeed, evidence has shown that the relative fructose content in the pooled soluble sugars (i.e. the sucrose/hexose ratio) plays a critical role in the pathogenesis of *Botrytis cinerea* (Lecompte *et al.*, 2017). In addition, changes in host carbon metabolism are important for the biotrophic adaptation of soil-borne pathogens (Kumar *et al.*, 2016; Ghosh *et al.*, 2017). The protein kinase complex SnRK1/TOR, which is homologous to SNF1 from *Saccharomyces cerevisiae*, is a central regulator of sugar metabolism (Grigston *et al.*, 2008; Halford and Hey, 2009). Interestingly, preliminary data showed that pepper SnRK1 was targeted by the effector AvrBsT, and that SnRK1 was required for plant immunity induced by the effector AvrBs1 (Szczesny *et al.*, 2010). In addition, AL2 from the tomato golden mosaic virus and BCTV L2 from the beet curly top virus can bind to and inactivate Arabidopsis SnRK1, thereby increasing plant susceptibility to Geminivirus infection (Hao *et al.*, 2003). Moreover, bacterial pathogens are known to regulate sugar efflux for nutritional gain via the delivery of TAL effectors using the type III secretory system (Xu *et al.*, 2019; Gupta, 2020). TAL ­effectors can enter plant cell nuclei, acting as transcription activators and binding to the effector-binding elements on the promoters of *SWEET* transporter genes, which normally function as dominant susceptibility (*S*) genes, thus leading to successful pathogen infection (Xu *et al.*, 2019; Chen *et al.*, 2020; Gupta, 2020). On the other hand, sugar and its by-products can act as key signals that activate the plant immune response against diverse pathogens (Gebauer *et al.*, 2017). Accumulating evidence suggests that sugar-signaling plays pivotal roles in plant disease resistance to various pathogens (Liang *et al.*, 2018; Yu *et al.*, 2022). Here, we found that VvRHIP1 might participate in the regulation of sugar signaling, acting in opposition to LtCre1 (Fig. 6). In the RNA-seq analysis of *N. benthamiana*, we found 242 shared DEGs in the *LtCre1*-overexpressing transgenic lines as compared to the WT (Supplementary Table S3; Supplementary Fig. S14). Interestingly, the ‘amino sugar and nucleotide sugar metabolism’ pathway was significantly enriched in these DEGs, and the glucose sensor hexokinase, which can regulate plant defense, was down-regulated in this pathway (Supplementary Fig. S15). These results therefore suggest that sugar signaling and sugar metabolism of the host plant might play a vital role in biotrophic adaptation during the grapevine–*L. theobromae* interaction. However, because the genetic transformation of grapevine is challenging, the changes in the sugar metabolism of the host plant at the *L. theobromae* infection site remain poorly characterized.

Previous studies have indicated that the post-translational regulation of host targets via multiple mechanisms is a characteristic virulence strategy utilized by fungal effectors (Li *et al.*, 2019). For example, the *Phytophthora* effector Avh241 has been shown to disrupt the self‐association of NDR1, thereby manipulating ETI‐associated cell death and promoting pathogen infection (Yang *et al.*, 2021). In rice, MoCDIP4 of the fungal pathogen *Magnaporthe oryzae* has been reported to competitively interact with the host mitochondria-associated OsDjA9 protein, weakening the association between it and the dynamin-related GTPase OsDRP1E protein, leading to accumulation of OsDRP1E and increased pathogen susceptibility (Xu *et al.*, 2020). In contrast, RXLR207 promotes the degradation of BPA1 and BPLs proteins, disrupting ACD11 stabilization through 26S proteasome-dependent degradation, and thus enhancing the defense response of the host plant (Li *et al.*, 2019). Here, we demonstrated that the effector LtCre1 competes with VvRHIP1 to bind to VvRGS1, thereby disrupting the association between VvRHIP1 and VvRGS1 (Fig. 6). However, details of the LtCre1 interference mechanisms remain unclear and need to be explored in further studies.

**Fig. 7.**
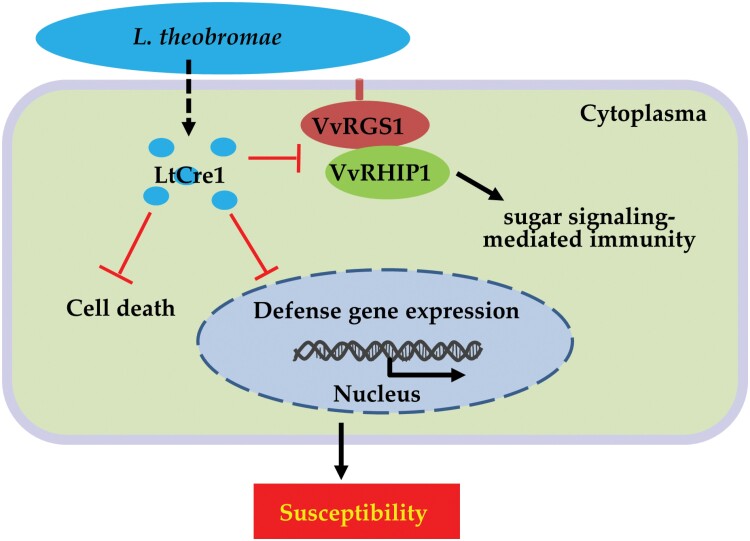
A proposed working model for the role of LtCre1 in disrupting plant immunity in grapevine. In the absence of *Lasiodiplodia theobromae* infection, VvRHIP1 acts as a positive regulator of plant sugar signaling and innate immunity. During *L. theobromae* infection, LtCre1 is translocated into the host plant cell, where it physically interacts with VvRHIP1 to competitively decrease its association with VvRGS1. The weakening of the VvRHIP1–VvRGS1 association interferes with sugar signaling and suppresses the expression of immune-response genes, and increases host susceptibility to *L. theobromae*.

Based on our results and those of previous studies, we propose a working model of the modulation of plant immunity by the *L. theobromae* effector LtCre1(Fig. 7). In the absence of infection, VvRHIP1 functions as a positive regulator of plant sugar signaling and innate immunity. During *L. theobromae* infection, LtCre1 is translocated into the host plant cell, where it physically interacts with VvRHIP1 to competitively decrease its association with VvRGS1. The weakening of the VvRHIP1–VvRGS1 association interferes with sugar signaling and suppresses the expression of genes related to plant immunity. In future work, we aim to characterize the molecular mechanisms underlying the balance between plant sugar signaling and innate immunity.

## Supplementary data

The following supplementary data are available at *JXB* online.

Table S1. Primers used in this study.

Table S2. Statistics of transcriptome sequencing of wild-type and transgenic *N. benthamiana*.

Table S3. Differentially expressed genes between wild-type *N. benthamiana* and transgenic lines overexpressing *LtCre1*.

Fig. S1. Characterization of phenotypes of *L. theobromae LtCre1*-overexpression and RNAi*LtCre1-*knockdown transformants.

Fig. S2. Phenotypes of transgenic *N. benthamiana* plants overexpressing *LtCre1*.

Fig. S3. Transcript levels of *NbPR1* and *NbLOX* in transgenic *N. benthamiana* overexpressing *LtCre1* after inoculation with *L. theobromae*, as determined using qRT-PCR with *NbTUBLIN6* as the reference gene.

Fig. S4. Transcript levels of PTI-associated genes in transgenic *N. benthamiana* overexpressing *LtCre1* after inoculation with *L. theobromae*.

Fig. S5. Sequence of the LtCre1 protein and its subcellular localization in leaf epidermal cells of transformed *N. benthamiana*.

Fig. S6. RT-PCR verification of the expression of *GFP*, *BAX*, and *LtCre1* in leaves of transformed *N. benthamiana*.

Fig. S7. Suppression of *Burkholderia gluma*e-triggered cell death by LtCre1 in leaves of transformed *N. benthamiana*.

Fig. S8. Multiple-sequence alignment of grapevine RHIP1 with RHIP homologs from other plant species.

Fig. S9. Split-luciferase complementation assay testing the interaction of LtCre1 with VvRHIP1.

Fig. S10. Expression of *VvRHIP1* in response to *L. theobromae* infection in grapevine and in transgenic *N. benthamiana* overexpressing *VvRHIP1*, and expression of *NbPR1* and *NbLOX* in transgenic *N. benthamiana* following infection.

Fig. S11. Transcript levels of the PTI-associated genes in transgenic *N. benthamiana* lines overexpressing *VvRHIP1* following *L. theobromae* infection.

Fig. S12. Sequence alignment of grapevine RGS1 with RGS1 homologs from Arabidopsis.

Fig. S13. Analysis of the interactions between VvRHIP1 and VvRGS1 using yeast two-hybrid assays.

Fig. S14. Analysis of DEGs between wild-type and transgenic *N. benthamiana* overexpressing *LtCre1*.

Fig. S15. Effects of *LtCre1* overexpression in *N. benthamiana* on the ‘amino sugar and nucleotide sugar metabolism’ pathway.

Fig. S16. Validation of six randomly selected DEGs by qRT-PCR using *NbEF1α* as the reference.

Fig. S17. Validation of six randomly selected DEGs by qRT-PCR using *NbTUBULIN6* as the reference.

erad055_suppl_Supplementary_Tables_S1-S2_Figures_S1-S17

erad055_suppl_Supplementary_Table_S3

## Data Availability

All data supporting the findings of this study are available within the paper and within its supplementary materials published online. All RNA-seq data produced in this study have been deposited in the NCBI GEO database (https://www.ncbi.nlm.nih.gov/geo/) under accession number GSE189162.
