# Supplementary material for: The woody plant-degrading pathogen Lasiodiplodia theobromae effector LtCre1 targets the grapevine sugar-signaling protein VvRHIP1 to suppress host immunity
Source: J Exp Bot. 2023 Feb 15;74(8):2768–85. doi: 10.1093/jxb/erad055 (PMC10112684; doi:10.1093/jxb/erad055)
Supplement: erad055_suppl_Supplementary_Tables_S1-S2_Figures_S1-S17 [file erad055_suppl_supplementary_tables_s1-s2_figures_s1-s17.pdf]

Supplementary Information

Supplementary Table S1 Primers used in this study.

Primers	Sequences	Purpose
Cre-EvF	AGATATCATGCTCGGTGTCCGCTTG	Construct overexpression vector for *Lasiodiplodia theobromae* transformation
Cre-PR	ACTGCAGTCCGACGAATTTACCCCCG	
CreRi-PF1	TCTGCAGGACCCCAAAGGGACGACAC	Construct RNAi vector for *Lasiodiplodia theobromae* transformation
CreRi-SR1	AGTCGACTTCCACGTGCTTGTCCGTC	
CreG-BIF	GGGATCCATGCTGATTTACTGCACCAAG	Construct Cam35S:LtCre1△SP-GFP vector for tobacco transformation
CreG-SIR	CGTCGACTCCGACGAATTTACCCCCGCAG	
CreSP-EF	CCGGAATTCATGCTCGGTGTCCGCTTG	Construct vector for yeast secretion assay
CreSP-XR	CCGCTCGAGCCTGCTTTCTTCAGGC	
CreB-SmF	ACCCGGGATGCTGATTTACTGCACCAAG	Construct vector for BAX-triggered cell death in tobacco
CreB-SIR	TGTCGACTTATCCGACGAATTTACCCCCG	
CreSI-F	AGTCGACCTGATTTACTGCACCAAG	Construct vector for *Burkholderia glumae*-triggered cell death in tobacco
CreBI-R	AGGATCCTCCGACGAATTTACCCCCG	
CreBi-BF	GCGGATCCATGCTGATTTACTGCACCAAG	Construct vector for BiFC
CreBi-SR	AGCCCGGGTCCGACGAATTTACCCCCGC	
RHIP1Bi-BF	CGGATCCATGGCGGAGGCGACGCCGTCG	Construct vector for BiFC
RHIP1Bi-SR	TCCCGGGAACAGGAACCCCTAATTCAG	
RHIPL-BF	TGGATCCATGGCGGAGGCGACGCCGTC	Construct vector

		for Split LUC
RHIPL-SR	AGTCGACAACAGGAACCCCTAATTCAG	
CreL-BF	TGGATCCCATGCTGATTTACTGCACCAAG	Construct vector for Split LUC
CreL-SR	AGTCGACTCATCCGACGAATTTACCC	
pMal-Cre1-F	TCGAGGGAAGGATTTCAGAAATGCTGATTTACTGC	Construct vector for pull down
pMal-Cre1-R	TCGACTCTAGAGGATCCGAATCATCCGACGAATTT	
pGEX-4T-RHIP-F	TTCCGCGTGGATCCCCGGAAATGGCGGAGGCGACG	Construct vector for pull down
pGEX-4T-RHIP-R	TCGAGTCGACCCGGCTAGAATCAAACAGGAACCCC	
pET28a-RGS-F	GCGGCCGCAAGCTTGTCGACTCATTGCTGCTCTAC	Construct vector for pull down
pET28a-RGSC-R	AGCAAATGGGTCGCGGATCCATGAAAATGAGCTTG	
pH35SC-Cre1-GFP-pF	GTCGACCTGCAGGCGGCCGCATGCTGATTTACTGC	Construct vector for CoIP
pH35SC-Cre1-GFP-pR	TCCTCGCCCTTGCTCACCATTCCGACGAATTTACC	
pH35SC-RHIP-myc-pF	GTCGACCTGCAGGCGGCCGCATGGCGGAGGCGACG	Construct vector for CoIP
pB2GW7m-RHIP-myc-pR	TCGACCCATGGGATCTACAGATCCTCTTCAGAGATGAGTTTCTGCTCAACAGGAACCCCTAA	
Cre-F	ATCCTCAAGCAGCGATACGG	RT-PCR
Cre-R	CTGACTGCTGTCACCGGATT	
BAX-F	ATCGATATGGACGGGTCCGGGGAG	RT-PCR
BAX-R	GTCGACTCAGCCCATCTTCTTCCAGATGG	
GFP-F	GACGTAAACGGCCACAAGTT	RT-PCR
GFP-R	CTCCAGCAGGACCATGTGAT	
Actin-F	AATCGTGAGGGATGTGAAGG	RT-PCR
Actin-R	GCATTTTCTGTGCACAATGG	
CreY-EIF	GGAATTCATGCTGATTTACTGCACCAAG	Y2H
CreY-SIR	CGTCGACTCCGACGAATTTACCCCCGCAG	
RHIP1Y-EF	CGGAATTCATGGCGGAGGCGACGCCGTCGTCAG	Y2H
RHIP1Y-XR	CCGCTCGAGAACAGGAACCCCTAATTCAGAAATC	
BD-RGS-EF	TGGCCATGGAGGCCGAATTCATGAAAATGAG	

	CTTGAGGAAA	Y2H
BD-RGS-SR	TGCGGCCGCTGCAGGTCGACTTGCTGCTCTACATCCTGGGT	
Cre-NF	AGCGGCCGCATGCTGATTTACTGCACCAAG	Y3H
Cre-BR	AAGGATCCTCCGACGAATTTACCC	
Cre-qF	CTCACATTCCTCGCTATC	qRT-PCR
Cre-qR	ACAAACTCCTCGTTCAAA	
LtActin-qF	GAAGGACCTGTACGGCAACA	qRT-PCR
LtActin-qR	AGGGCGGTGATTTCCTTCTG	
LtTubulin-qF	AATCGGTGCTGCTTTCTGG	qRT-PCR
LtTubulin-qR	TTGTTGGACGCCTCGTTG	
NbEF1 α-qF	AAGGTCCAGTATGCCTGGGTGCTTGAC	qRT-PCR
NbEF1 α-qR	AAGAATTCACAGGGACAGTTCCAATACCA	
NbTubulin6-qF	CTTCTCACGCATTGACCATA	qRT-PCR
NbTubulin6-qR	TCATCTTCGTCTTCTCCCTC	
NbPR1-qF	CCGCCTTCCCTCAACTCAAC	qRT-PCR
NbPR1-qR	GCACAACCAAGACGTACTGAG	
NbLOX-qF	AAAACCTATGCCTCAAGAAC	qRT-PCR
NbLOX-qR	ACTGCTGCATAGGCTTTGG	
NbPti5-qF	CCTCCAAGTTTGAGCTCGGATAGT	qRT-PCR
NbPti5-qR	CCAAGAAATTCTCCATGCACTCTGTC	
NbAcre31-qF	AATTCGGCCATCGTGATCTTGGTC	qRT-PCR
NbAcre31-qR	GAGAAACTGGGATTGCCTGAAGGA	
NbGras2-qF	TACCTAGCACCAAGCAGATGCAGA	qRT-PCR
NbGras2-qR	TCATGAGGCGTTACTCGGAGCATT	
RHIP1-qF	CACTGGAAGTCAACTCAAG	qRT-PCR
RHIP1-qR	GATGTCATAGAAGCAACCTC	
VvEF1 γ-F	GCGGGCAAGAGATACCTCAA	qRT-PCR
VvEF1 γ-R	TCAATCTGTCTAGGAAAGGAAG	
VvEF1α-qF	AGGAGGCAGCCAACTTCACC	qRT-PCR
VvEF1α-qR	CAAACCCTGCATCACCATTC	
NbTBL26-qF	GGTTATGTAGATGAGGTGATG	qRT-PCR
NbTBL26-qR	GCTGATATTGCCTGTAGAC	
Niben101Scf00698g00008-qF	ACGCTAATCTATTCAAGT	qRT-PCR
Niben101Scf00698g00008-qR	TGAGTAGTCCAGTATGAT	
Niben101Scf04894g00006-qF	GTATGATGACAGTGACAAGAG	qRT-PCR
Niben101Scf048	TGGTATGTGAGTGTGGATT	

94g00006-qR		
Niben101Scf11214g00007-qF	CGATGATTCTAAGCGTGAG	qRT-PCR
Niben101Scf11214g00007-qR	CAGGTCCATAGTTGAAGTTC	
Niben101Scf09672g01005-qF	GTAGTGAAGGTATGTGATGTAAT	qRT-PCR
Niben101Scf09672g01005-qR	CTGAATAAGGTGTAACTTGTGTA	
Niben101Scf02011g04003-qF	CCAACAACCTTATGCCATT	qRT-PCR
Niben101Scf02011g04003-qR	GCTATGTGTCCATCTGCTA	
Niben101Scf02417g06009-qF	AGAGGTCTGTGATGTTGT	qRT-PCR
Niben101Scf02417g06009-qR	CTTCTACTGTAATAATGCTAATCC	

Supplementary Table S2 Statistics of transcriptome sequencing.

Sample	CK	OV3	OV10	OV14
Total reads	45270250	40283428	40164524	41013938
Total mapped	41105884 (90.8%)	37029588 (91.92%)	38151270 (94.99%)	38944623 (94.95%)
Multiple mapped	4074340 (9%)	3043647 (7.56%)	3546199 (8.83%)	3737991 (9.11%)
Uniquely mapped	37031544 (81.8%)	33985941 (84.37%)	34605071 (86.16%)	35206632 (85.84%)
Read-1	18395986 (40.64%)	17032856 (42.28%)	17250398 (42.95%)	17542709 (42.77%)
Read-2	18635558 (41.17%)	16953085 (42.08%)	17354673 (43.21%)	17663923 (43.07%)
Reads map to '+'	18497984 (40.86%)	16989288 (42.17%)	17293368 (43.06%)	17586086 (42.88%)
Reads map to '-'	18533560 (40.94%)	16996653 (42.19%)	17311703 (43.1%)	17620546 (42.96%)

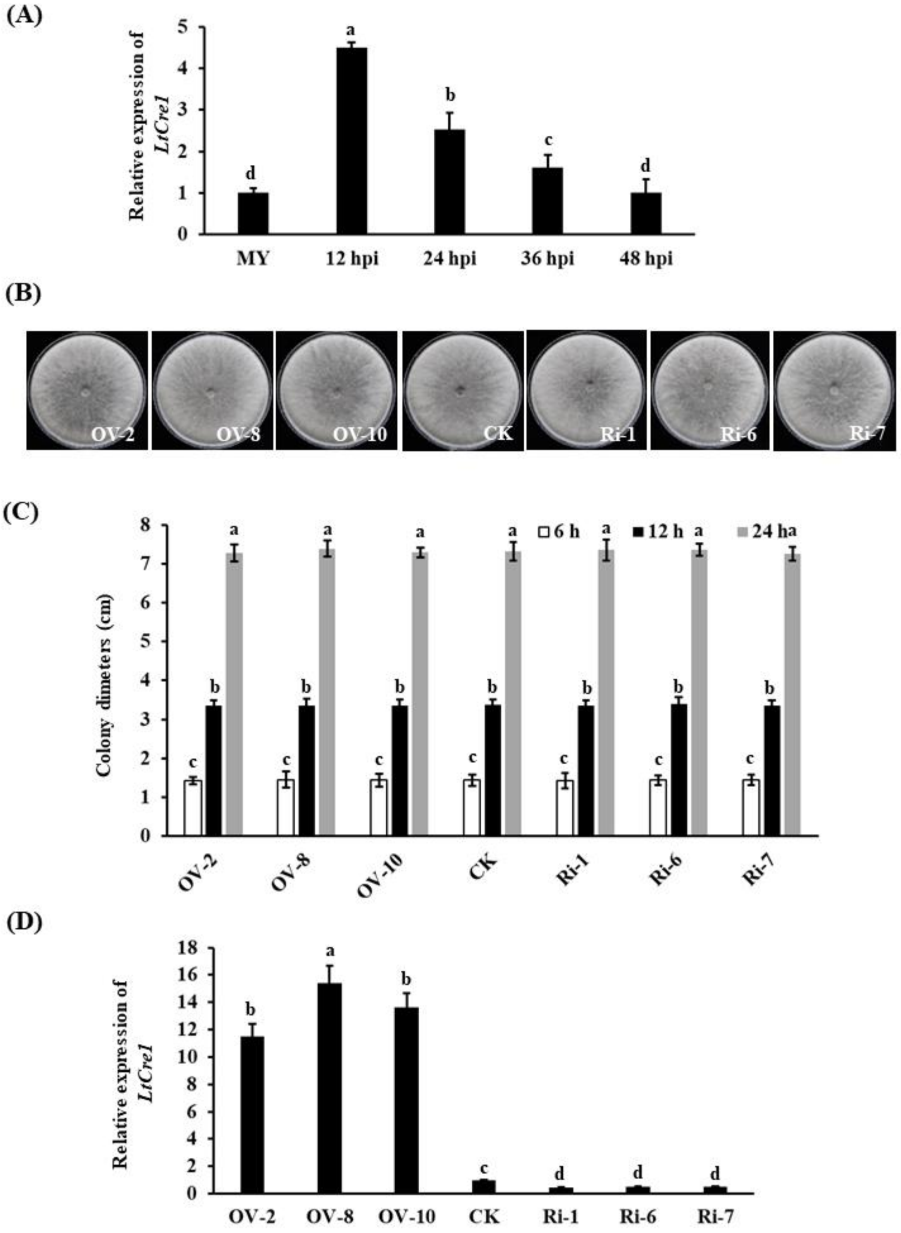

Supplementary Fig. S1 Phenotype observation of *Lasiodiplodia theobromae* *LtCre1*-overexpression transformants and RNAi-mediated *LtCre1*-knockout transformants. (A) The relative expression patterns of the effector gene *LtCre1* gene during *L. theobromae* infection, as determined using qRT-PCR. The *LtTubulin* gene of *L. theobromae* was used as the internal reference gene for transcript normalization. (B) The morphology of *LtCre1* overexpression transformants (OV-2, OV-8, and OV-10) and RNAi-mediated *LtCre1*-knockout transformants

(Ri-1, Ri-6 and Ri-7). Colonies were cultured on PDA plates for 6 days at 26°C. (C)The colony growth rates of *LtCre1*-overexpression transformants and RNAi-mediated *LtCre1*-knockout transformants. The transformants were cultured on PDA medium, with eight replicated plates per strain. Then the diameter for each colony was measured. (D) Relative expression levels of the *LtCre1* gene in *L. theobromae LtCre1*-overexpression transformants (OV-2, -8, and -10) and RNAi-mediated *LtCre1*-knockout transformants (RI-1, -6, and -7), as determined using qRT-PCR. The *LtTubulin* gene of *L. theobromae* was used as the internal reference gene for transcript normalization. Different lowercase letters above the bars indicate significant differences ($p < 0.05$); Duncan's multiple range test.

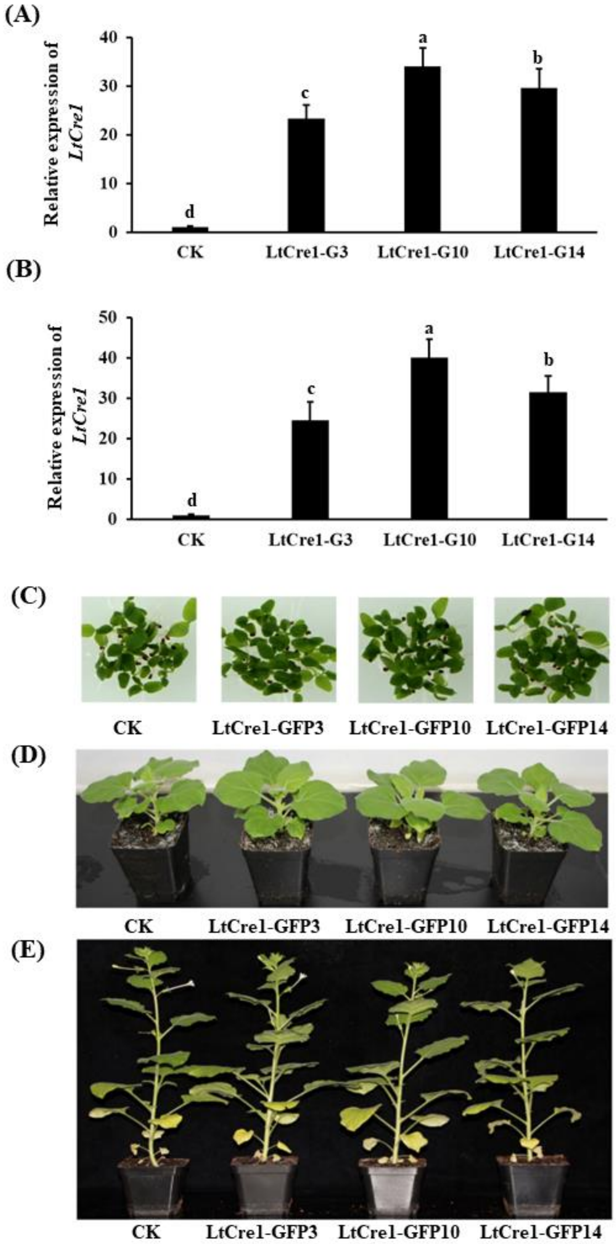

Supplementary Fig. S2 Phenotypes of the transgenic *Nicotiana benthamiana* plants. (A) Relative transcript abundance of *LtCre1* in the leaves of transgenic *N. benthamiana* overexpressing *LtCre1* (T3 generation; LtCre1-GFP3, LtCre1-GFP10, LtCre1-GFP14) as compared to the *N. benthamiana* wild type (CK). *NbEF1*α of *N. benthamiana* was used as the internal reference gene for normalization. (B) The relative transcript abundance of *LtCre1* in the leaves of transgenic *N. benthamiana* plants. The *NbTubulin6* of *N. benthamiana* was used as the internal reference gene for normalization. (C) Seed germination of *N. benthamiana* plants on 1/2 MS medium ten days

after growing. (D-E) The development phenotype of the transgenic *N. benthamiana* lines at four (D) and seven weeks (E) after planting.

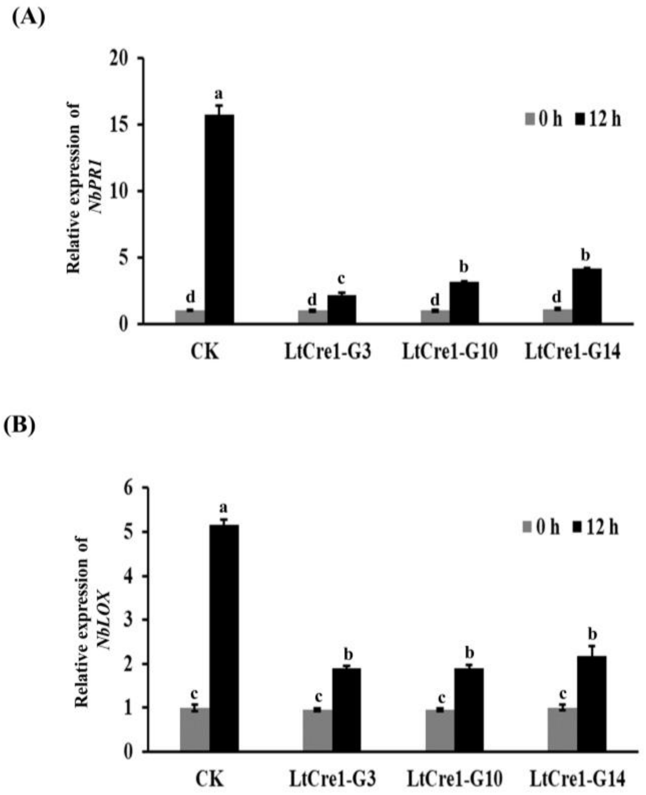

Supplementary Fig. S3 The transcript levels of the defense-related genes *NbPR1* (A) and *NbLOX* (B) in transgenic *Nicotiana benthamiana* overexpressing *LtCre1* 12 h after inoculation with *L. theobromae*, as revealed by qRT-PCR. The *NbTubulin6* of *N. benthamiana* was used as the internal reference gene.

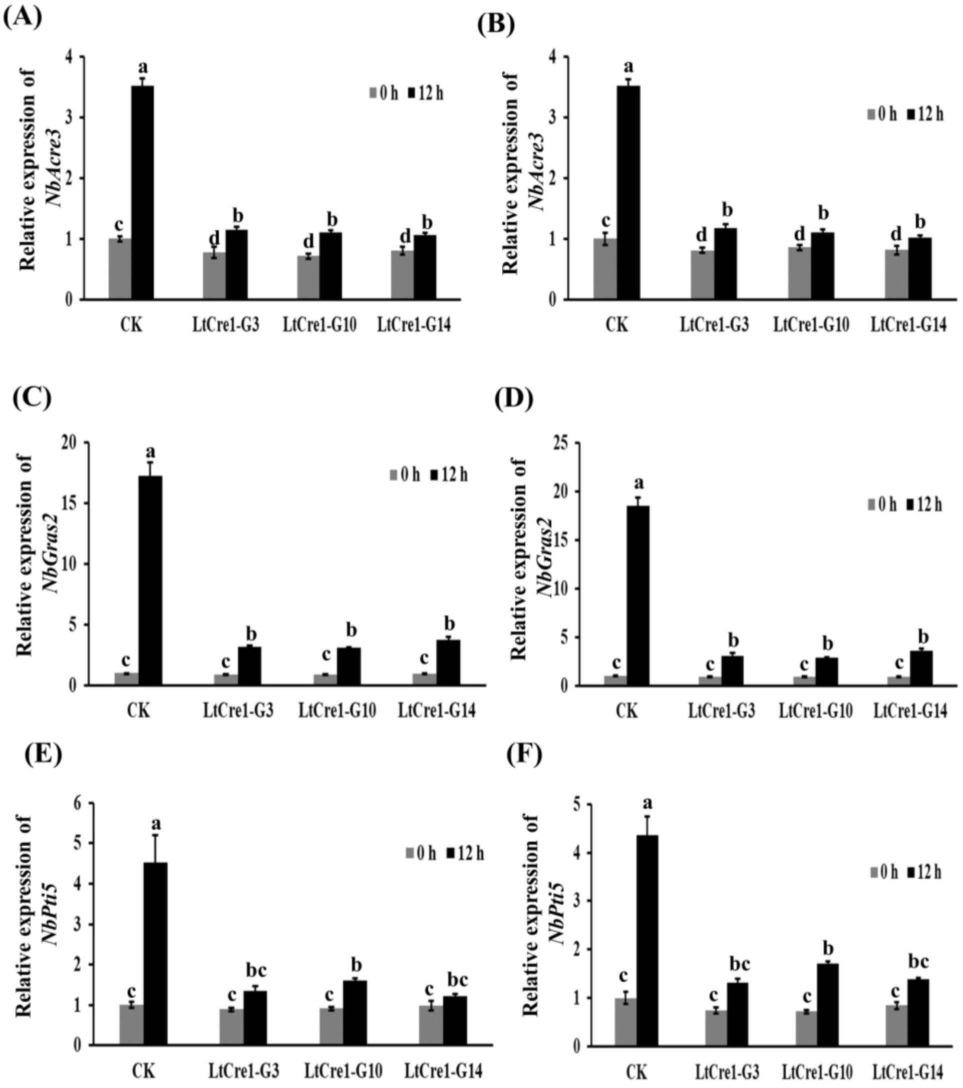

Supplementary Fig. S4 The transcript levels of PTI-associated genes in transgenic *Nicotiana benthamiana* overexpressing *LtCre1* after *Lasiodiplodia theobromae* infection. The transcript levels of *NbAcre3* (A and B), *NbGras2* (C and D) and *NbPti5* (E and F) in transgenic *N. benthamiana* overexpressing *LtCre1* as compared to the *N. benthamiana* wild type upon flg22 treatment, as revealed by qRT-PCR. In panels A, C and E, expression levels were normalized against the *NbEF1*α gene of *N. benthamiana*, while in panels B, D and F, expression levels were normalized against the the *NbTubulin6* gene.

(A)

MLGVRLLTCLIDLSAEALIYCTKTWLEELPEESRAESAVRSTIL
EQFWRALGLSNDHDDDPVVGYPEARADGLDLEERTSLAQAL
RRAVYYNDRSILKQRYGRLLTLFFDPKGTTQSDDAPAIQKLVT
EVLKLPRSSYNDSEASRKIRTVYSSMLPWLSDAETDGMDDGA
VETSTDKHVEGQQEGILPSTEANVTAGEECEICDDSIGFESFAW
ARCGQGHEFVRCSLTFLAIQAPGISKLCGICGKRYLNEEFVVRT
DKLAGAAEARQSGDSSQQDVVMPDADGGSSKQGGSTGQDR
APAVDYGRSPITLARILFAACDVCIYCGGKFVG

(B)

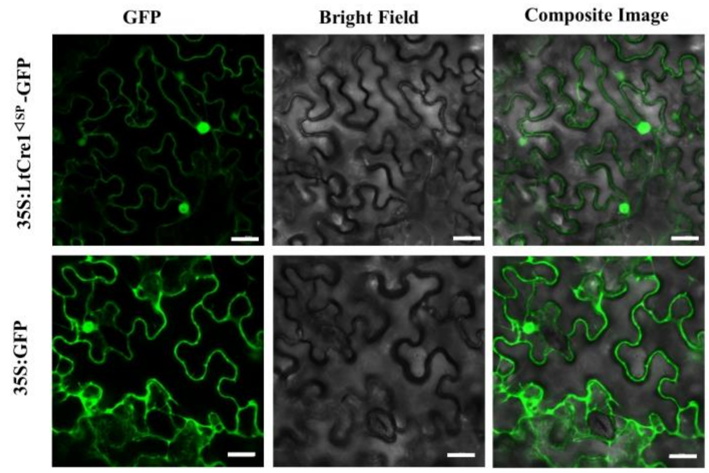

Supplementary Fig. S5 LtCre1 is predicted to be a secreted protein. (A) *LtCre1* encodes a 331-amino acid protein. The green characters represent the putative N-terminal secretion signal peptide (SP, 1-17 aa), and the red characters indicate the RxLG-dEER motif. (B) Subcellular localization of LtCre1 in *Nicotiana benthamiana* epidermal cells. The expression of the LtCre1$^{\triangle SP}$-GFP fusion protein was observed under a confocal laser-scanning microscope 48 h post-agroinfiltration. LtCre1$^{\triangle SP}$-GFP was localized exclusively in the nucleus and the cytoplasm. Images from left to right: GFP, bright-field, and merged. GFP, green fluorescent protein. Scale bars = 20 μm.

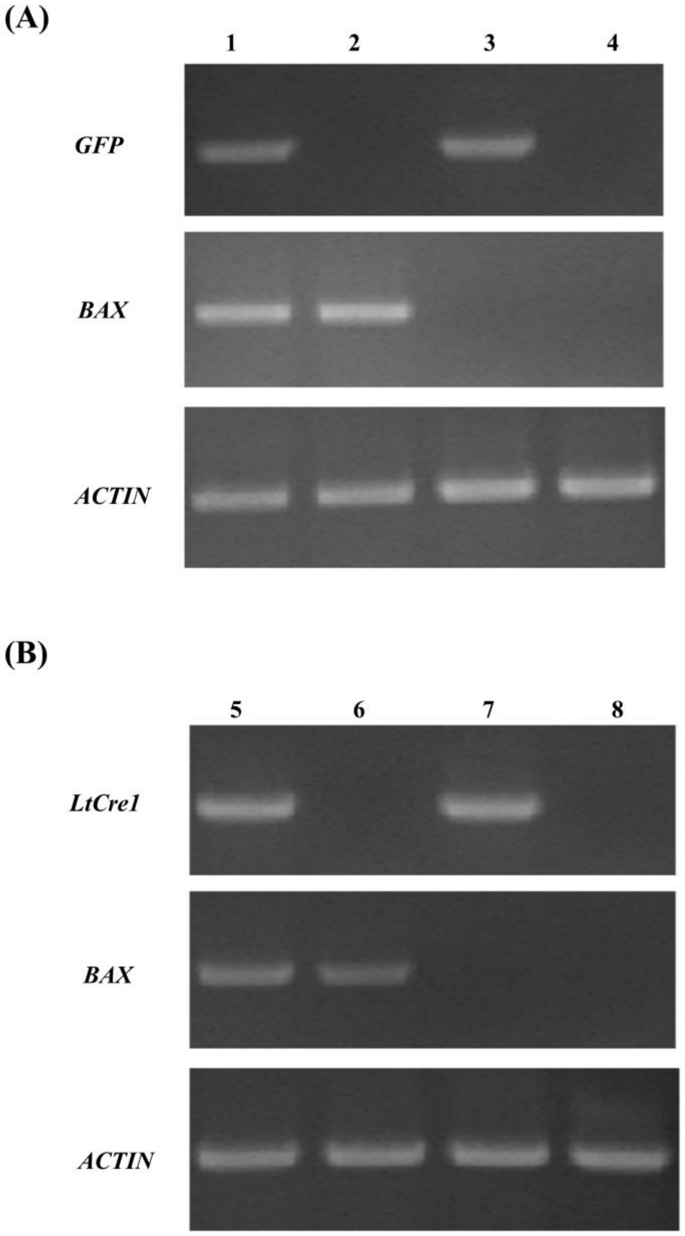

Supplementary Fig. S6 RT-PCR verification of the expression of *GFP*, *BAX*, and *LtCre1* in *Nicotiana benthamiana* leaves.

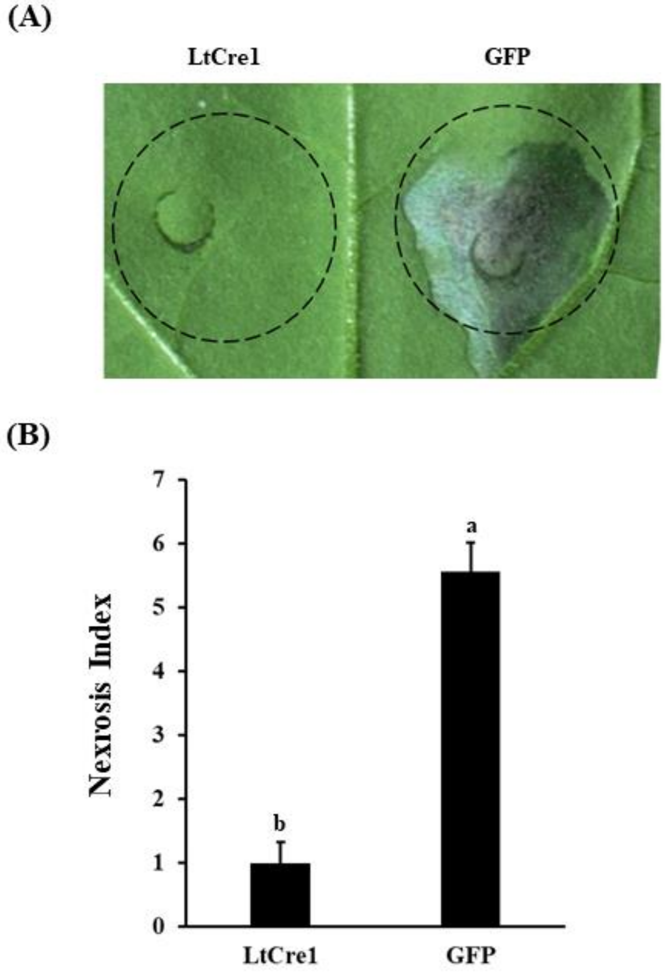

Supplementary Fig. S7 Suppression of *Burkholderia glumae*-triggered cell death in *Nicotiana benthamiana* leaves by LtCre1. Four-week-old *N. benthamiana* leaves were infiltrated with buffer or *Agrobacterium tumefaciens* cells containing the *LtCre1* gene. Photos of the phenotypes were taken 5 days post *Agrobacterium* infiltration.

```
AtRHIP1.pro   MSETEATGVTDDSAPAIETETVSDAMEHTAIGVVESVEGAIEGAEKWVGDLQRTVKESKD   60
NbRHIP1.pro   MGEEAP........PAVDIDPSSSSLPLPSQNKEKAWHSYIS......EDLPRTVQESTD   46
VvRHIP1.pro   MAEATP...........SSAPAPADGESISQQQQKPWHISFA......EDLQRTVSESAD   43
Consensus     m e                                              dl rtv es d

AtRHIP1.pro   TAMRSARSLRENSTSQFRSIQDFIPHALTQYKTYENAFFSKVTDELIYAKEHPAAAIGIG   120
NbRHIP1.pro   SALRSARSIQYTSSTHLRTLQDFMPKIKAQYTTYEEVFFRKIKDELVTAREHPAMAGGIG   106
VvRHIP1.pro   SAIRSALSLQQNSSSHLRSLQEFIPQMESQYRTYEDAFFKKVKDELTSAKEHPVVVGAVA   103
Consensus      a rsa s    s    r  q f p    qy tye  ff k  del  a ehp

AtRHIP1.pro   VAASLVLMRGPRRFLFRNTLGRFQSEEAQFLKAEKHVQELNMSVDLMKKESRKLLERTAL   180
NbRHIP1.pro   IAAGLLLMRGPRRFLFRQTLGRLQSEEAKFSKAEKNVKELSLSVDLMKKESKKLLERAAL   166
VvRHIP1.pro   VTAGLIFLRGPRRFLFHHTLGRFQSEEAQFVRAEKNVKELNLSVDLMKNESRKLLERAAL   163
Consensus       a l   rgprrflf  tlgr qseea f  aek v el  svdlmk es kller al

AtRHIP1.pro   AEKDMKRGLSELMNSGNDIHRLAKSVHKAECEAADLMDGLRQIPGRDAIKLRAEVASMTS   240
NbRHIP1.pro   AEKDMKRGLSDLMDAGNQIQSIGKTVYKVEAQAADLMDVLREIPGREALKLRAEVASMAS   226
VvRHIP1.pro   AEKDMKCGHTELMNTGSQLKRLAKTVFKVEAQAADLMDGLRETPGREALKLRSEVASMTS   223
Consensus     aekdmk g   lm  g       k v k e  aadlmd lr  pgr a klr evasm s

AtRHIP1.pro   LIRQKRIALNKRIMGMSDLGVS                                         262
NbRHIP1.pro   HLRQQRTAIDKRIVKVSELGVP                                         248
VvRHIP1.pro   LIKQQRIALDKRIMKISELGVP                                         245
Consensus      l q r a  kri   s lgv
```

Supplementary Fig. S8 Multiple sequence alignment of grapevine RHIP1 with RHIP homologs from various other plant species. The identical amino acids are highlighted in green. At, *Arabidopsis thaliana*; Nt, *Nicotiana benthamiana*; Vv, *Vitis vinifera*.

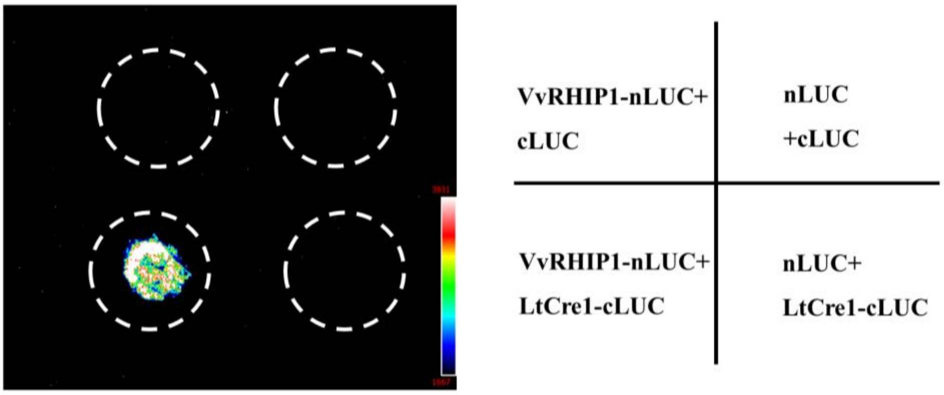

Supplementary Fig. S9 Split-luciferase complementation assay testing the interaction of LtCre1 with VvRHIP1. *Agrobacterium* containing the combined vectors were infiltrated into the leaf cells of four-week-old *N. benthamiana* plants. The fluorescence signal was detected 48 h later.

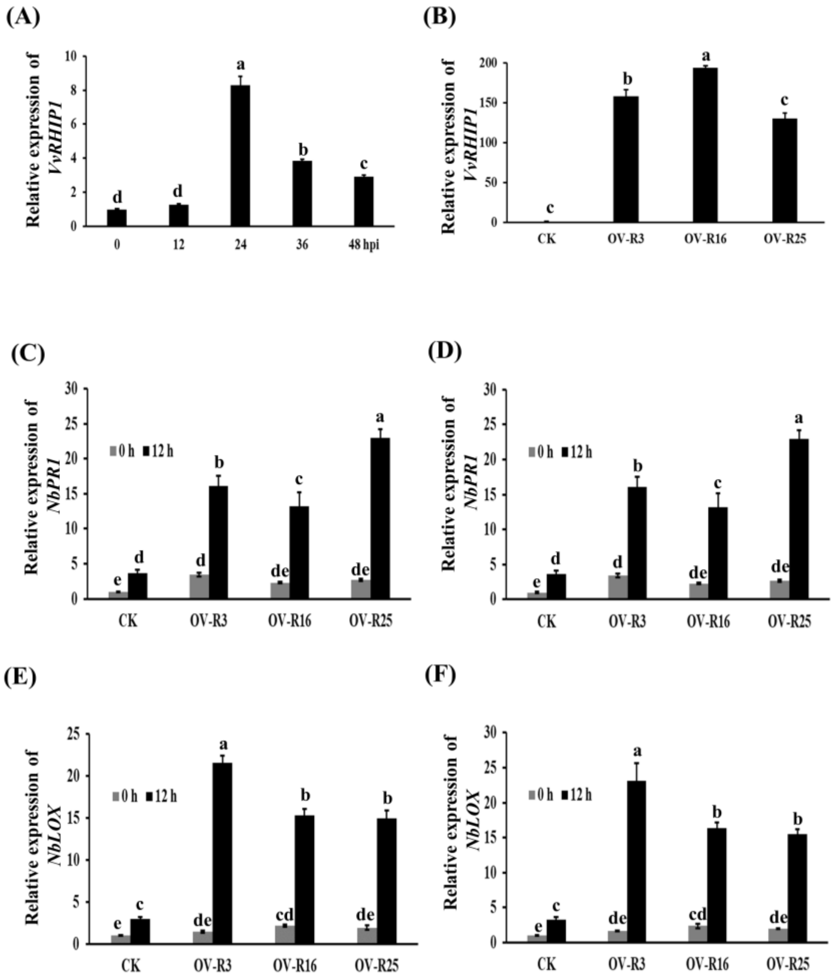

Supplementary Fig. S10 VvRHIP1 positively regulates plant immunity. (A) Relative expression profile of *VvRHIP1* during *L. theobromae* infection, as determined by qRT-PCR. The *VvEF1α* gene of *Vitis vinifera* was used as the internal reference gene. (B) Relative transcript abundance of *VvRHIP1* in transgenic *N. benthamiana* lines overexpressing *VvRHIP1* (OV-R3, OV-R16, and OV-R25). *NbEF1*α was used as the internal reference gene for normalization. (C−F) qRT-PCR quantification of the relative transcript levels of *NbPR1* (C and D) and *NbLOX* (E and F) in transgenic *N. benthamiana* lines overexpressing *VvRHIP1* at 12 hours post inoculation with *L. theobromae*. In panels C and E, expression levels were normalized to *NbEF1*a, while in panels C and E, the *NbTubulin6* gene was used as the internal reference gene.

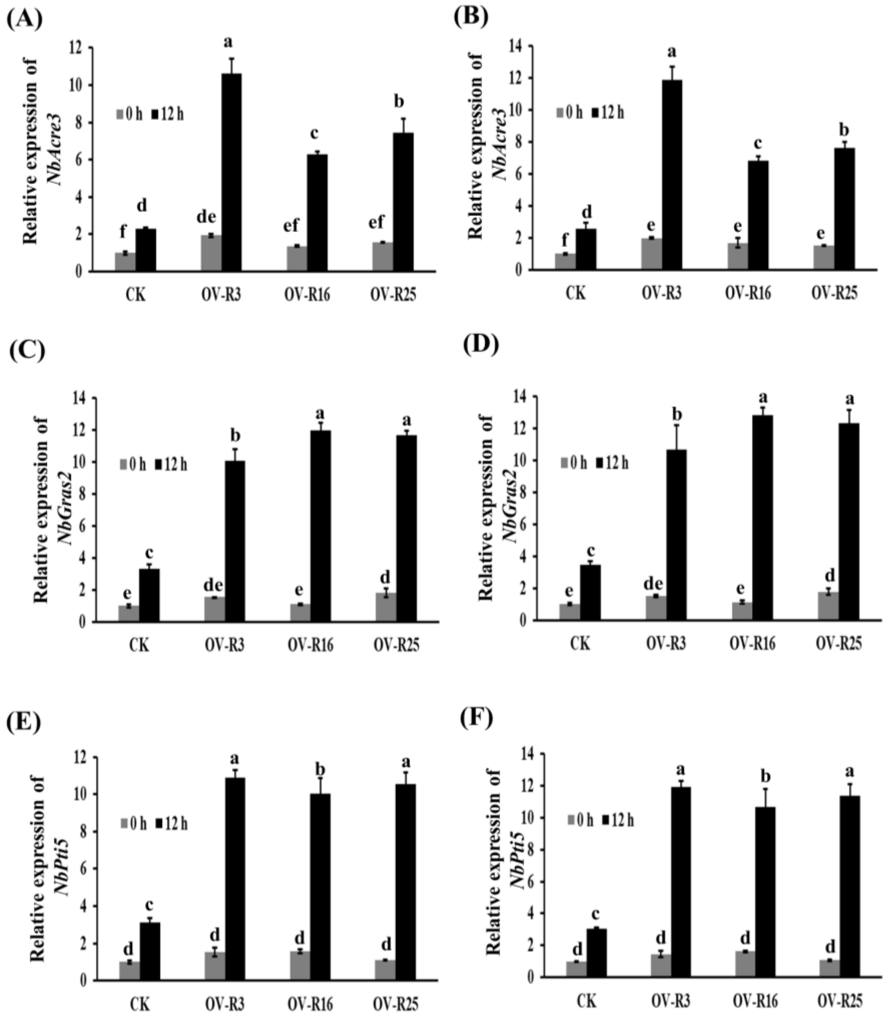

Supplementary Fig. S11 Transcript levels of the PTI-associated genes in transgenic *Nicotiana benthamiana* lines overexpressing *VvRHIP1* upon *Lasiodiplodia theobromae* infection. The relative transcript levels of *NbAcre3* (A and B), *NbGras2* (C and D) and *NbPti5* (E and F) in transgenic *N. benthamiana* lines overexpressing *VvRHIP1* (OV-R3, OV-R16, and OV-R25) upon flg22 treatment, as revealed by qRT-PCR. In panels A, C and E, gene expression levels were normalized to the *NbEF1*α gene of *N. benthamiana*, while in panels B, D and F, gene expression levels were normalized to the *NbTubulin6* gene. The error bars represent the standard deviation (SD) of three independently replicated experiments. In all graphs, different lowercase letters above the bars indicate significant differences ($p < 0.05$; Duncan's multiple range test).

```
VvRGS1.pro   MGS.CGKNGGCPSDYIAVAISLLCFILLIIKATLPFIVHKVPRPKGSGFWIPVIQVFASL    59
AtRGS1.pro   MASGCALHGGCPSDYVAVAISVICFFVLLSRSVLPCLIHKAPRTNSSSFWIPVIQVISSF    60
Consensus    m s c   ggcpsdy avais  cf  ll    lp l hk pr   s fwipviqv  s

VvRGS1.pro   NLILSIVMSINFLKFKKKHWWQSCYLWAVWVEGPLGFGLILSCRIVQAFQLYYIFVKRRL   119
AtRGS1.pro   NLIFSIMMSVNLIRFRTKHWWRYCYLWAVWIEGPLGFGLIMSCRITQAFQLYFIFVKKRL   120
Consensus    nll si ms n l f  khww  cylwavw egplgfgll scri qafqly ifvk rl

VvRGS1.pro   PPIRSYVFLPTIVLPWIAGAALIHKKKPLNERCHLGTRWIIPVVLLHTTYVAALVGFTVA   179
AtRGS1.pro   PPVKSYIFLPLVLLPWIFGAAIIHATKPLNDKCHMGLQWTFPVAGLHALYVLALIAFTRA   180
Consensus    pp  sy flp   lpwi gaa  h  kpln  ch g  w  pv  lh  yv al  ft a

VvRGS1.pro   IRHIEFRFHELKDLWRGILVSTSSVGLWVTAYILNEIHDDIEWLQVTSRFLLIIMASILV   239
AtRGS1.pro   VRHVEFRFDELRDLWKGILVSATSIVIWVTAFVLNEIHEEISWLQVASRFVLIVTGGILV   240
Consensus     rh efrf el dlw gilvs  s   wvta  lneih  i wlqv srf ll    ilv

VvRGS1.pro   LAFFSMSSSQPLLSKMSLRKREATEFETMGRALGIPDSGLLLQREPAPDIDPNEPLDKLL   299
AtRGS1.pro   VVFFSISSNQPLLSQISLKKRQNFEFQRMGQALGIPDSGLLFRKEEFRPVDPNEPLDKLL   300
Consensus      ffs ss qplls  sl kr   ef  mg algipdsgll   e     dpnepldkll

VvRGS1.pro   LNKGFRQSFMAFADSCLAGESVHFYDEVHELAKIPVDDFVRRIYMARHIIEKYVIAGATM   359
AtRGS1.pro   LNKRFRHSFMEFADSCYAGETLHFFEEVYEHGKIPEDDSIRRIYMARHIMEKFIVAGAEM   360
Consensus    lnk fr sfm fadsc age  hf  ev e  kip dd  rriymarhi ek   aga m

VvRGS1.pro   EVNISHRNRQEILTTPDLAHPDLFNNALNELLQLMKMNLAKDYWSSMFFKKLREDGTGSN   419
AtRGS1.pro   ELNLSHKTRQEILTTQDLTHTDLFKNALNEVMQLIKMNLVRDYWSSIYFIKFKEE...ES   417
Consensus    e n sh  rqeiltt dl h dlf nalne  ql kmnl  dywss  f k  e

VvRGS1.pro   GHELEVVTGWNYS.PRLSCVHGEDDPFHEEHPSKSSAHDTNTQDVEQ                 465
AtRGS1.pro   CHEAMHKEGYSFSSPRLSSVQGSDDPFYQEHMSKSSRCSSPG.....                 459
Consensus     he     g    s prls v g ddpf  eh skss
```

Supplementary Fig. S12 Sequence alignment of grapevine RGS1 with RGS1 homologs from *A. thaliana*. The identical amino acids are highlighted in green. Vv, *Vitis vinifera*; At, *Arabidopsis thaliana*.

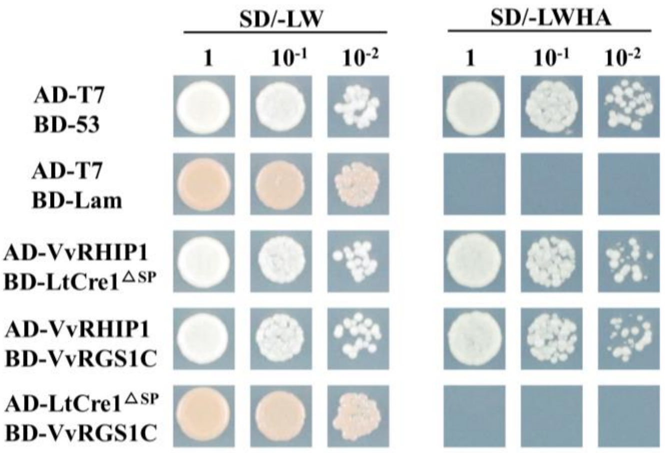

Supplementary Fig. S13 Analysis of the interactions between VvRHIP1 and VvRGS1 using yeast two-hybrid assays.

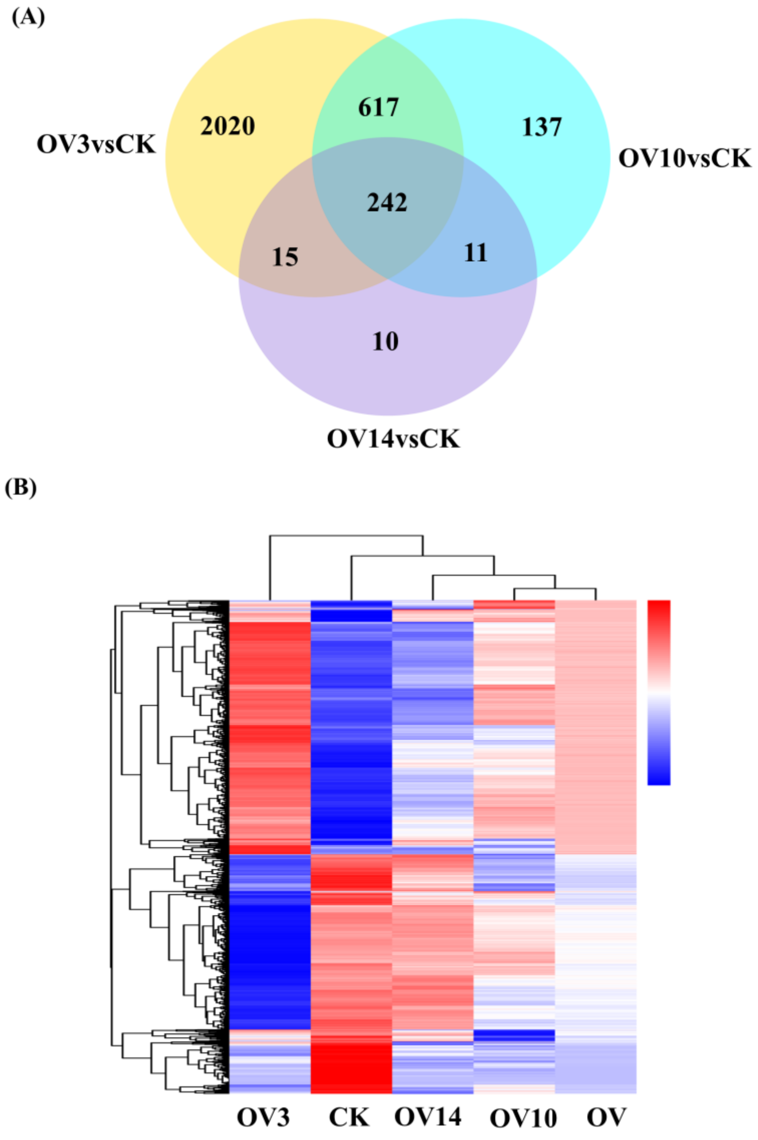

Supplementary Fig. S14 Statistical analysis of DEGs between wild type and transgenic *N. benthamiana* overexpressing *LtCre1*. (A) Venn diagram analysis showing transcripts distribution in wild type and transgenic *N. benthamiana* overexpressing *LtCre1*. (B) The numbers of upregulated and downregulated DEGs between wild type and transgenic *N. benthamiana* overexpressing *LtCre1*. CK, wild type *N. benthamiana* plants; OV3, transgenic *N. benthamiana* overexpressing *LtCre1* line LtCre1-G3; OV10, transgenic *N. benthamiana* overexpressing *LtCre1* line LtCre1-G10; OV14 transgenic *N. benthamiana* overexpressing *LtCre1* line LtCre1-G14.

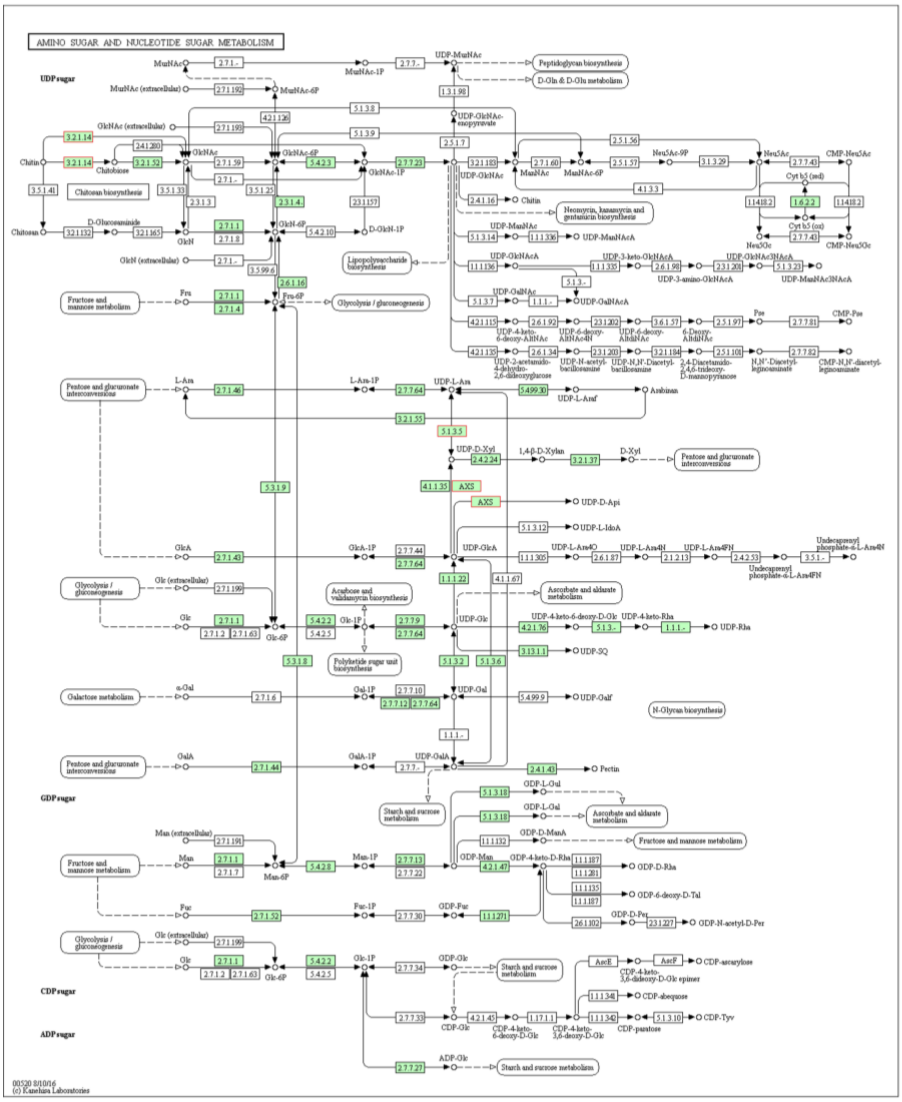

Supplementary Fig. S15. Effect of *LtCre1* overexpression on amino sugar and nucleotide sugar metabolism pathway of *N. benthamiana*. Red square indicates up-regulated expression.

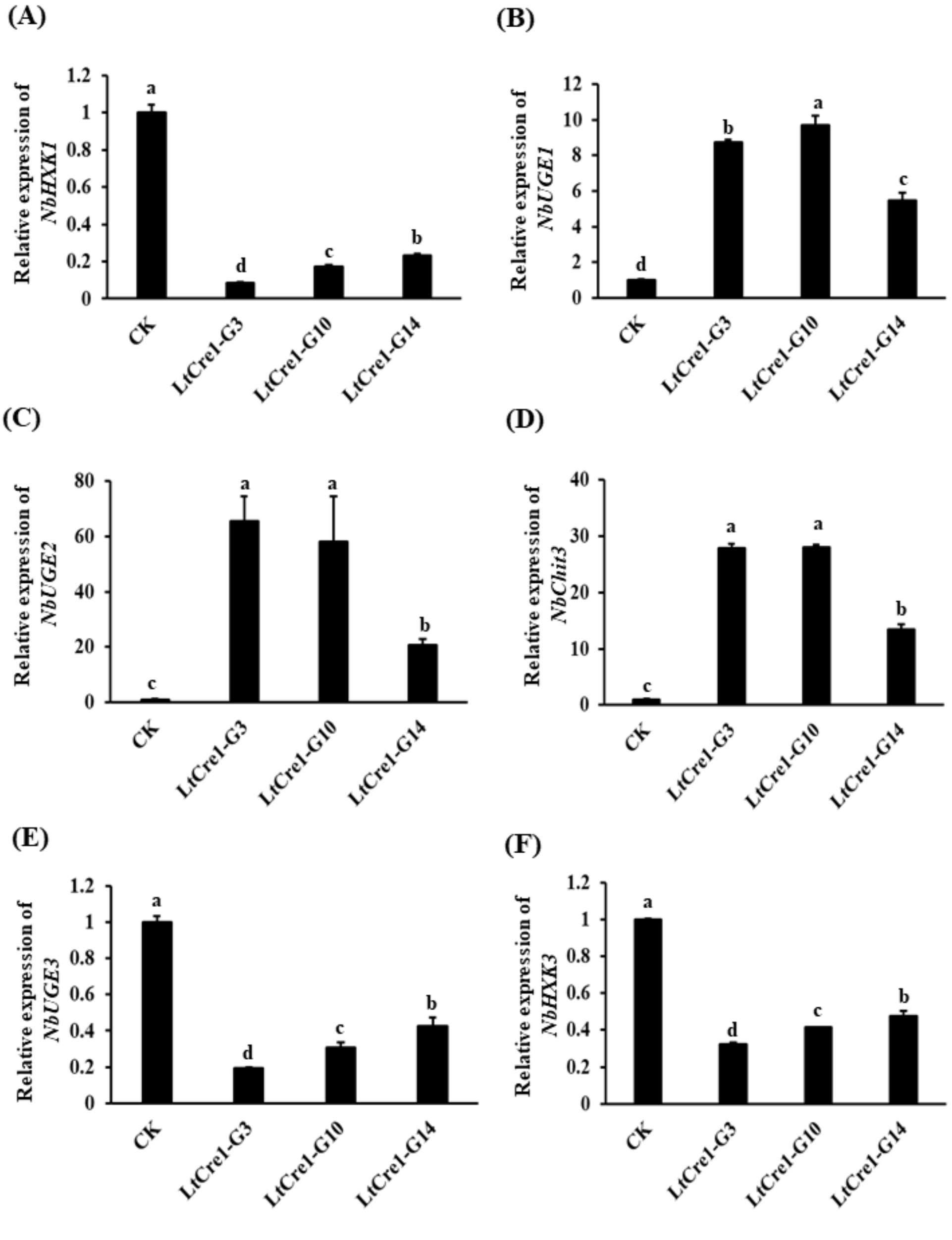

Supplementary Fig. S16 Validation of six randomly selected DEGs using qRT-PCR. The *N. benthamiana NbEF1*α gene was used for normalization. In all graphs, different lowercase letters above the bars indicate significant differences ($p < 0.05$; Duncan's multiple range test).

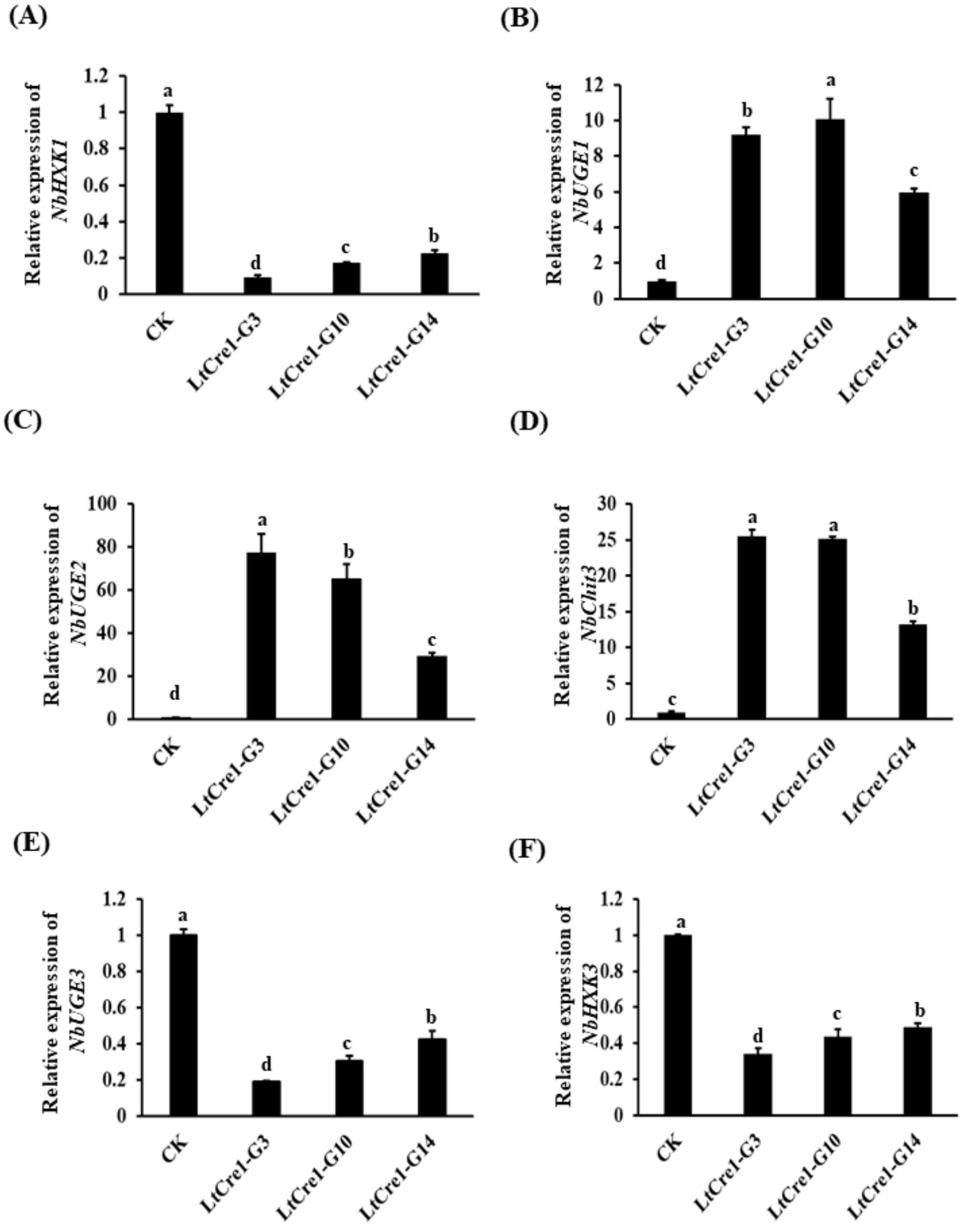

Supplementary Fig. S17 Validation of six randomly selected DEGs using qRT-PCR. The *N. benthamiana NbTubulin6* gene was used for normalization.
